# Cytokine and autoantibody clusters interaction in systemic lupus erythematosus

**DOI:** 10.1186/s12967-017-1345-y

**Published:** 2017-11-25

**Authors:** Yovana Pacheco, Julián Barahona-Correa, Diana M. Monsalve, Yeny Acosta-Ampudia, Manuel Rojas, Yhojan Rodríguez, Juliana Saavedra, Mónica Rodríguez-Jiménez, Rubén D. Mantilla, Carolina Ramírez-Santana, Nicolás Molano-González, Juan-Manuel Anaya

**Affiliations:** Center for Autoimmune Diseases Research (CREA) School of Medicine and Health Sciences, Universidad del Rosario, Carrera 26 # 63B-51, Bogota, Colombia

**Keywords:** Personalized medicine, Autoantibodies, Cytokines, Systemic lupus erythematosus, Subphenotypes, Cluster analysis, Antiphospholipid antibodies, Anti-dsDNA antibodies, Interleukin 8, Interferon alpha, Interleukin 12p40, Taxonomy

## Abstract

**Background:**

Evidence supports the existence of different subphenotypes in systemic lupus erythematosus (SLE) and the pivotal role of cytokines and autoantibodies, which interact in a highly complex network. Thus, understanding how these complex nonlinear processes are connected and observed in real-life settings is a major challenge. Cluster approaches may assist in the identification of these subphenotypes, which represent such a phenomenon, and may contribute to the development of personalized medicine. Therefore, the relationship between autoantibody and cytokine clusters in SLE was analyzed.

**Methods:**

This was an exploratory study in which 67 consecutive women with established SLE were assessed. Clinical characteristics including disease activity, a 14-autoantibody profile, and a panel of 15 serum cytokines were measured simultaneously. Mixed-cluster methodology and bivariate analyses were used to define autoantibody and cytokine clusters and to identify associations between them and related variables.

**Results:**

First, three clusters of autoantibodies were defined: (1) neutral, (2) antiphospholipid antibodies (APLA)-dominant, and (3) anti-dsDNA/ENA-dominant. Second, eight cytokines showed levels above the threshold thus making possible to find 4 clusters: (1) neutral, (2) chemotactic, (3) G-CSF dominant, and (4) IFNα/Pro-inflammatory. Furthermore, the disease activity was associated with cytokine clusters, which, in turn, were associated with autoantibody clusters. Finally, when all biomarkers were included, three clusters were found: (1) neutral, (2) chemotactic/APLA, and (3) IFN/dsDNA, which were also associated with disease activity.

**Conclusion:**

These results support the existence of three SLE cytokine-autoantibody driven subphenotypes. They encourage the practice of personalized medicine, and support proof-of-concept studies.

**Electronic supplementary material:**

The online version of this article (10.1186/s12967-017-1345-y) contains supplementary material, which is available to authorized users.

## Background

Systemic lupus erythematosus (SLE) is a heterogeneous systemic autoimmune disease (AD) characterized by a wide range of clinical and serological manifestations and a high disease burden [1]. SLE pathophysiology encompasses several mechanisms, such as T cell and B-cell abnormalities, impaired apoptotic debris clearance, autoantibody production, and abnormal cytokine secretion [2]. The diversity in clinical expression associated with different autoantibodies among patients supports the existence of different subphenotypes although similar treatment is given to almost all the patients with diverse effectiveness [2].

Autoantibodies are essential biomarkers for the diagnosis and classification of ADs, and several are known to be pivotal in the ADs pathophysiology [3]. In SLE they can form immune complexes, which may be deposited in tissues, and activate a direct immune response against a specific organ [4]. Autoantibodies are usually found long before symptom onset [5].

Cluster methodology of autoantibodies in SLE has been used to evaluate several cohorts, both adult and pediatric, worldwide and has given insight into the different subphenotypes due to the correlation among clusters, clinical features, and disease activity [6–17].

Autoimmune diseases evince similar immunopathogenic mechanisms (i.e., the autoimmune tautology) [18]. This explains the fact that one AD may coexist with one or more ADs (i.e., polyautoimmunity) [19], and that one AD may carry several autoantibodies with diverse specificity. Polyautoimmunity has been observed in up to 40% of patients with SLE [20, 21]. In addition, non-lupus autoantibodies are observed frequently in SLE patients. Rheumatoid factor (RF) and anti-cyclic citrullinated peptide (CCP) antibodies are present in 42 and 5.6% respectively, but only 6.4% of the patients meet the criteria for rhupus [22]. Antiphospholipid antibodies (APLA) may be present in 54% of patients although antiphospholipid syndrome (APS) develops in only 10% of SLE patients [23]. In euthyroid patients with SLE, anti-thyroid peroxidase antibodies (TPOAb) and anti-thyroglobulin antibodies (TgAb) are observed in 21 and 10% of patients respectively, but confirmed autoimmune hypothyroidism is diagnosed in 12% [24].

Cytokines play an essential role in the pathophysiology of SLE. Thus, a number of cytokine-targeted therapies which have shown promising results have been developed, particularly in some subphenotypes of the disease [25]. However, the immune system shows a wide variation at both intra- and inter-individual levels. These differences among individuals, which may explain the differences observed among patients, have been called “immunotypes” [26].

Since human biology is a complex set of interacting components that work together to produce an outcome, a system approach may elucidate these interactions [27]. Systems biology of human disease, also known as systems medicine or network medicine, aims at identifying the main components of a system and at measuring how they change when the system is disturbed [26]. Since understanding the connections of the nonlinear complex processes of cytokines and autoantibodies in real-life settings is a major challenge, we analyzed the simultaneous relationship between them in patients with established SLE.

## Methods

### Study population

This was a cross-sectional analytical study of 67 consecutive women with SLE. The subjects have been systematically followed at the Center for Autoimmune Diseases Research (CREA) in Bogota, Colombia. All the subjects fulfilled the 1997 update of the American College of Rheumatology (ACR) classification criteria for SLE [28]. The patient socio-demographic and cumulative clinical and laboratory data were obtained by interview, standardized report form, physical examination and chart review as previously reported [23, 29]. The data were collected in an electronic and secure database.

### Clinical variables

Clinical and laboratory variables were registered as present or absent at any time during the course of the disease as previously reported [23]. Other manifestations such as polyautoimmunity [19, 30] and current pharmacological treatment were also assessed.

Current disease activity was measured using the Systemic Lupus Activity Questionnaire (SLAQ), a well-known Patient-Reported Outcome (PRO) tool, which presents an adequate performance in large community-based cohorts [31–33]. As SLAQ is unavailable in Spanish, a linguistic validation was done. Working independently, two English proficient physicians (JBC, MR) translated the original US English version into Spanish [32]. Afterwards, they worked together to obtain a single Spanish version. A mother tongue professional translator independently back-translated this version into an English one. Lastly, the physicians compared the two versions to produce a second Spanish version. If there was disagreement, a third English- proficient physician (YR) decided which was the best version. Finally, a definite Spanish form was acquired and used with the patients (see Additional file 1).

### Laboratory measurements

Serum samples were obtained during a state of fasting. A total of 14 autoantibodies were evaluated in the sera of patients. Detection of IgM RF, IgG anti-CCP third-generation (CCP3), IgM and IgG anti-cardiolipin antibodies (ACA), IgM and IgG anti-β2glycoprotein-1 (β2GP1) antibodies, IgG anti-double-stranded DNA (dsDNA) antibodies, IgG TgAb, and TPOAb were all quantified by the Enzyme-Linked-Immunosorbent Assay (ELISA) as previously reported [24]. Antinuclear antibodies (ANAs) were evaluated by using an indirect immunofluorescence assay. Positive ANA were considered from dilution 1/80. Negative and positive controls, provided by the manufacturer, were analyzed in parallel. Anti-Ro, anti-La, anti-RNP, and anti-Sm were also evaluated by ELISA. All the assay kits were from Inova Diagnostics, Inc. (San Diego, CA, USA).

Concentration of 15 human cytokines (IL-2, IL-10, IL-6, IL-8, IL-9, IL-13, IL-12/23p40, G-CSF, IFNγ, IFNα, IL-4, IL-1β, TNFα, IL-5, IL-17A) in serum samples from patients was assessed by Cytometric Bead Array (CBA, Becton–Dickinson Biosciences, San Diego, CA, USA). The test was done in accordance with the manufacturer’s protocols. Briefly, 50 µL of assay beads and 50 µL of the sample under study or standard were added to each sample tube. The samples were incubated at room temperature in the dark for 1 h. Next, the samples were washed with 1 mL of wash buffer, centrifuged, and the resulting pellet was resuspended in 50 µL of PE-labeled antibodies (Detection Reagent). The samples were further incubated for 2 h, washed again, and centrifuged. After discarding the supernatant, the pellet was resuspended in 300 µL of wash buffer and analyzed on the same day in a FACSCanto II™ flow cytometer (BD Bioscience™). Before the analysis, the cytometer was standardized using calibration beads in accordance with the manufacturer’s protocol. For each cytokine, a standard curve was assessed, and concentration of each cytokine was calculated as an interpolation of the standard curve using the FCAP Array™ Software (BD Bioscience™). Results were considered positive when the assay results were above a threshold value, which was confirmed in healthy individuals in whom evidence of acute or chronic disease including autoimmune, cardiovascular, or metabolic was not detected (Table 3) [34, 35].

### Statistical analyses

The mixed-cluster methodology proposed by Lebart et al. [36] was used to find groups of patients with similar autoantibody and cytokine profiles. In short, cluster analysis seeks groups of individuals with similar values across several variables. The number of groups is algorithmically determined and consolidated in two steps: first, a hierarchical cluster analysis is done based on Ward’s distance, for which the number of clusters is determined by means of the between-cluster inertia gain criterion. Second, the cluster membership for each individual is consolidated using a k-means algorithm on the centroids of each cluster. In the end, a categorical variable in which each individual is assigned to one and only one of the clusters derived is obtained [36]. Afterwards, a description of each cluster is developed by studying the distribution of each of the original variables used for clustering in each of the derived groups. This determine the composition and relation of the original variables and the clusters obtained.

This clustering method was used to obtain autoantibody clusters (named profiles from here on) based on the 14 autoantibodies, and cytokine profiles based on the 15 cytokines measured. Cytokines and autoantibodies with frequencies under 5% were excluded from the cluster analysis, since variables with low frequencies tend to generate clusters of patients with such atypical results exclusively. To assess associations between abovementioned profiles and other variables, we used the Chi square and Kruskall–Wallis tests. Statistical analyses were done using R version 3.3.2.

### Ethics

This research was carried out in accordance with Resolution number 008430 of 1993 issued by the Ministry of Health of the Republic of Colombia and was classified as minimal risk research. The Ethics Committee of Universidad del Rosario approved the present project.

## Results

### Patients

The demographic, clinical, and laboratory characteristics of the patients are shown in Table 1. The median age of patients was 50 (38–57) years with a median age at SLE onset of 29 (22–40) years and a disease duration of 13 (9–21) years. Lupus nephritis was seen in 25 (37%) patients at diagnosis. At the time of the study, median disease activity by SLAQ was 16 (10.5–26.5). In addition, patients were receiving medication in the following numbers: 41 (61%) were receiving antimalarials, 39 (58%) corticosteroids, 20 (30%) azathioprine, 10 (15%) methotrexate, 8 (12%) mycophenolate mofetil, 4 (6%) rituximab, and 2 (3%) were receiving belimumab, leflunomide, sulfasalazine, and tacrolimus. No patient was receiving cyclophosphamide. The antibodies that were positive most frequently at the time of the study were ANAs (85%) followed by anti-dsDNA (48%) (Table 2). The cytokines that were positive most frequently were IL-12/23p40 (52%), G-CSF (46%), and IFNα (25%) (Table 3).

**Table 1:** General characteristics of 67 women with SLE

Age (IQR), years	50 (38–57)
Age at onset disease (IQR), years	29 (22–40)
Disease duration (IQR), years	13 (9–21)
Polyautoimmunity^a^ (%)	14 (21)
Educational level (%)	
< 9 years	10 (15)
≥ 9 years	57 (85)
Socioeconomic status^b^ (%)	
Low	19 (28)
Intermediate	37 (55)
High	11 (17)
1997 ACR Criteria at diagnosis (%)	
Positive ANAs	58 (87)
Immunologic criteria	54 (81)
Hematologic criteria	48 (72)
Non-erosive arthritis	47 (70)
Photosensitivity	41 (61)
Malar rash	33 (49)
Renal criteria^c^	25 (37)
Oral ulcers	23 (34)
Serositis	19 (28)
Neurologic criteria	12 (18)
Discoid rash	7 (10)

*ACR* American College of Rheumatology

^a^Polyautoimmunity signifies the presence of more than one autoimmune disease in a single patient

^b^Socioeconomic status was categorized based on Colombian legislation as previously reported [23, 24]

^c^Renal criteria was defined as active urinary sediment, or proteinuria > 500 mg/24 h or positive renal biopsy [23, 24]

**Table 2:** Autoantibodies in 67 patients with SLE at the time of the study

Autoantibody	N (%)
ANAs	57 (85)
dsDNA	32 (48)
Ro	26 (39)
RNP	25 (37)
RF	24 (36)
ACA-IgG	12 (18)
Sm	12 (18)
ACA-IgM	11 (16)
TPOAb	7 (10)
β2GP1-IgM	7 (10)
β2GP1-IgG	6 (9)
La	5 (7)
TgAb	5 (7)
CCP3	1 (1)

*ANAs* antinuclear antibodies, *dsDNA* anti-double stranded DNA antibodies, *RF* rheumatoid factor,ACA anticardiolipin antibody, *TPOAb* anti-thyroperoxidase antibody, *TgAb* anti-thyroglobulin antibody, *β2GP1* β2 glycoprotein-1, *CCP3* anti-cyclic citrullinated peptide third-generation

**Table 3:** Cytokine concentration in women with SLE

Cytokine	Healthy controls N = 5^a^	SLE patients N = 67^a^	Number of positive patients (%)^b^
IL-12/23p40	16.13 (18.9)	27.11 (48.9)	35/67 (52)
G-CSF	0 (0)	2.16 (6.19)	31/67 (46)
IFNα	0 (0)	3.72 (12.2)	17/67 (25)
IL-8	11.71 (4.5)	12.67 (25.1)	16/67 (24)
IL-6	0.11 (0.21)	4.99 (28.09)	15/67 (22)
IL-10	0 (0)	0.57 (1.79)	14/67 (21)
IL-1β	0 (0)	0.97 (4.66)	6/67 (9)
IL-17A	0 (0)	7.41 (33.9)	11/67 (16)
TNFα	0 (0)	2.11 (9.34)	9/67 (13)
IL-5	0 (0)	0.17 (0.77)	6/67 (9)
IL-4	0 (0)	0.39 (2.01)	4/67 (6)
IFNγ	0 (0)	0.39 (2.1)	4/67 (6)
IL-2	0 (0)	0.39 (2.23)	2/67 (3)
IL-9	0 (0)	0.13 (0.75)	2/67 (3)
IL-13	0 (0)	0.02 (0.19)	1/67 (1.5)

*IL* interleukin, *G-CSF* granulocyte colony-stimulating factor, *IFN* interferon, *TNF* tumor necrosis factor

^a^Mean (SD), in pg/mL

^b^Data correspond to those patients with positive values as compared to healthy controls (above the threshold) [34, 35]

### Autoantibody clusters

**Fig. 1:**
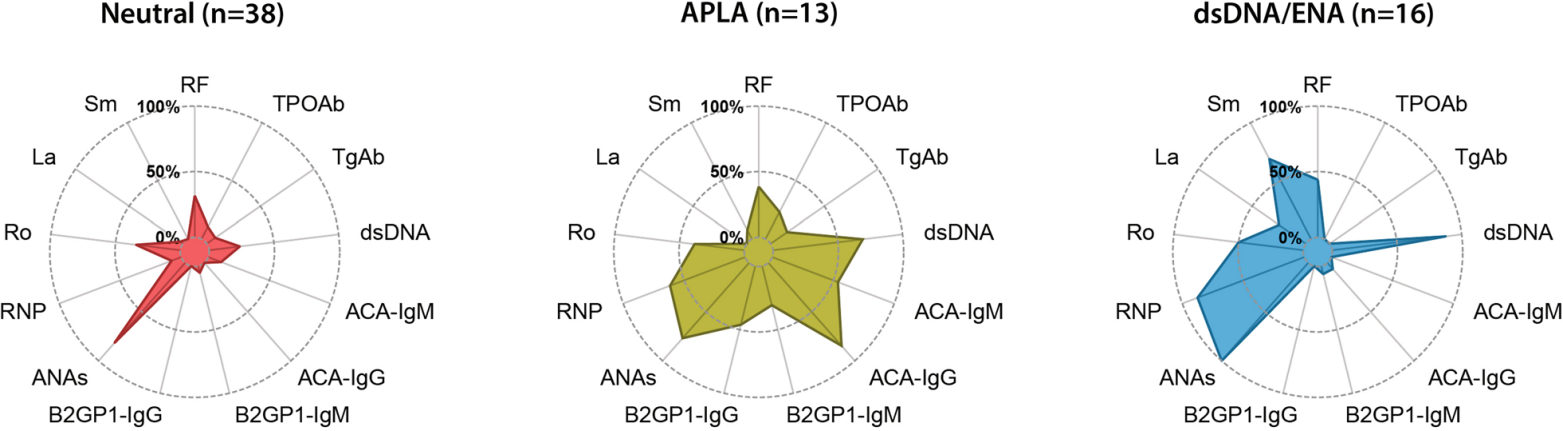
Autoantibody clusters

Three clusters of autoantibodies were defined (Fig. 1): (1) neutral, in which the frequency of specific autoantibodies other than ANAs was below 40%; (2) APLA-dominant, which showed a greater than 50% frequency of ACA-IgG/IgM, anti-dsDNA, and anti-RNP; and (3) anti-dsDNA/ENA-dominant, which presented a high frequency of anti-dsDNA, anti-RNP, and anti-Sm antibodies. Table 4 shows the distribution of autoantibodies within clusters. No association between autoantibody clusters and disease activity was found.

**Table 4:** Distribution of autoantibodies among autoantibody clusters

Autoantibody	Neutral (n = 38)	APLA-dominant (n = 13)	dsDNA/ENA-dominant (n = 16)	p-value
ANAs	31 (82)	10 (77)	16 (100)	0.145
RF	12 (32)	5 (38)	7 (44)	0.678
CCP3	1 (3)	0 (0)	0 (0)	0.678
TPOAb	4 (10)	3 (23)	0 (0)	0.129
TgAb	3 (8)	2 (15)	0 (0)	0.289
dsDNA	9 (24)	9 (69)	14 (87)	< 0.0001*
ACA-IgM	4 (10)	7 (54)	0 (0)	0.0002*
ACA-IgG	0 (0)	11 (85)	1 (6)	< 0.0001*
β2GP1-IgM	2 (5)	4 (31)	1 (6)	0.028
β2GP1-IgG	0 (0)	6 (46)	0 (0)	< 0.0001*
RNP	3 (8)	8 (61)	14 (87)	< 0.0001*
Ro	13 (34)	5 (38)	8 (50)	0.553
La	1 (3)	0 (0)	4 (25)	0.008
Sm	0 (0)	1 (8)	11 (69)	< 0.0001*

Data correspond to number of patients (%)

*ANAs* antinuclear antibodies, *RF* rheumatoid factor, *CCP3* anti-cyclic citrullinated peptide third-generation, *TPOAb* anti-thyroperoxidase antibody, *TgAb* anti-thyroglobulin antibody, *dsDNA* anti-double stranded DNA antibodies, *ACA* anticardiolipin antibody, *β2GP1* β2 glycoprotein-1

* Statistically significant after Bonferroni correction

### Cytokine clusters

**Fig. 2:**
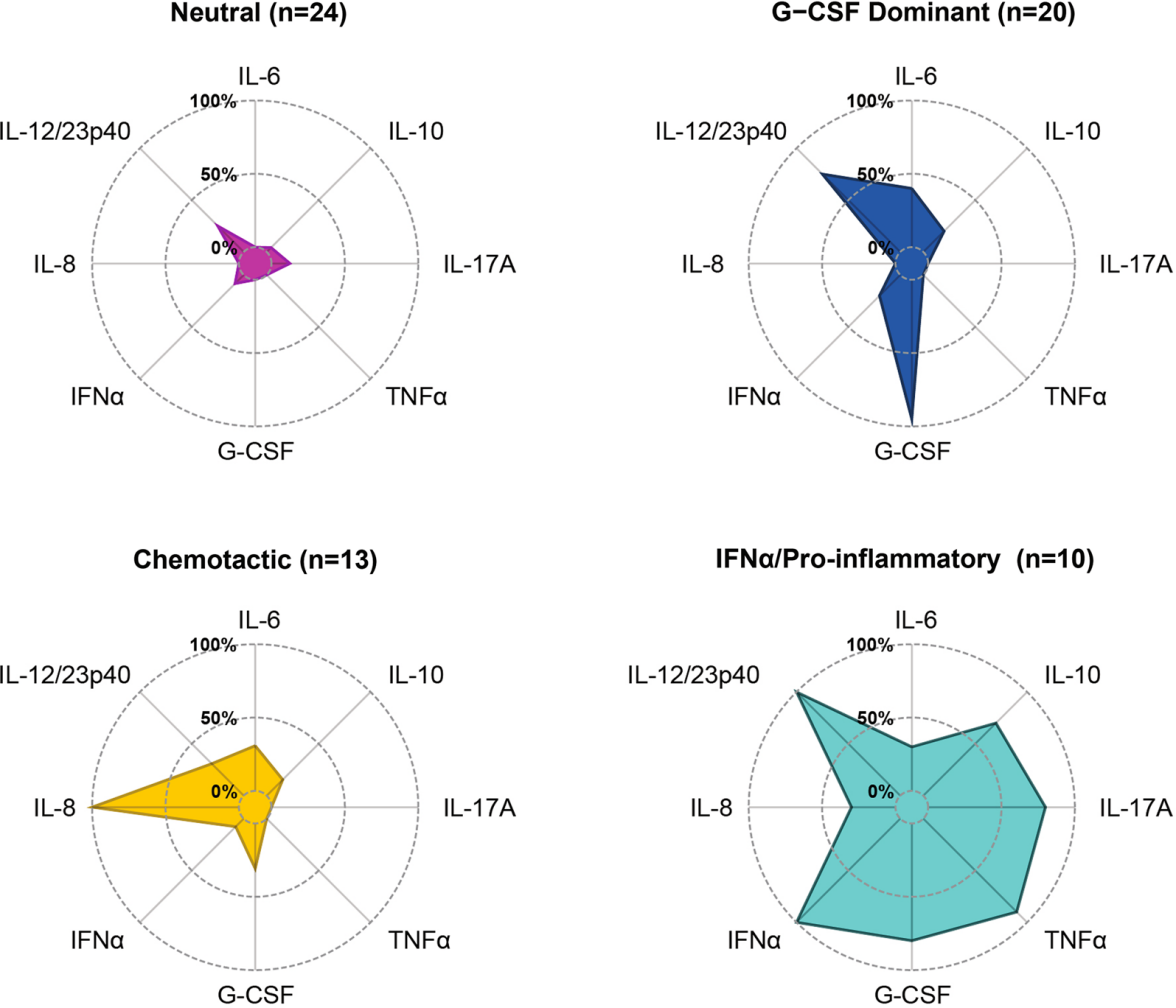
Cytokine clusters

Eight cytokines showed levels above the threshold (i.e., > 5%) (Table 3). Four clusters were defined (Fig. 2): (1) neutral, which exhibited a low frequency of cytokines; (2) chemotactic, characterized by a predominance of IL-8; (3) G-CSF dominant, which presented a high frequency of G-CSF, and IL-12/23p40; and (4) IFNα/Pro-inflammatory, which was dominated by the presence of IFNα, IL-12/23p40, TNFα, IL-17A, G-CSF, and IL-10. Table 5 shows the distribution of cytokines within clusters.

**Table 5:** Distribution of cytokines among cytokine clusters

Cytokine	Neutral (n = 24)	Chemotactic (n = 13)	G-CSF (n = 20)	IFNα/Pro-inflammatory (n = 10)	p-value
IL-2	1 (4)	0 (0)	0 (0)	1 (10)	0.418
IL-4	0 (0)	0 (0)	1 (5)	3 (30)	0.005
IL-5	1 (4)	0 (0)	1 (5)	4 (40)	0.002*
IL-6	0 (0)	4 (31)	8 (40)	3 (30)	0.009
IL-9	1 (4)	0 (0)	0 (0)	1 (10)	0.418
IL-10	1 (4)	2 (15)	4 (20)	7 (70)	0.0003*
IL-17A	3 (12)	0 (0)	0 (0)	8 (80)	< 0.0001*
TNFα	0 (0)	0 (0)	0 (0)	9 (90)	< 0.0001*
G-CSF	0 (0)	4 (31)	19 (95)	8 (80)	< 0.0001*
IFNα	2 (8)	1 (8)	4 (20)	10 (100)	< 0.0001*
IFNγ	0 (0)	1 (8)	1 (5)	2 (20)	0.162
IL-13	0 (0)	0 (0)	0 (0)	1 (10)	0.122
IL-1β	0 (0)	1 (8)	0 (0)	5 (50)	< 0.0001*
IL-8	0 (0)	13 (100)	0 (0)	3 (30)	< 0.0001*
IL-12/23p40	6 (25)	4 (31)	15 (75)	10 (100)	< 0.0001*

Data correspond to number of positive patients (%)

*IL* interleukin, *G-CSF* granulocyte colony-stimulating factor, *IFN* interferon, *TNF* tumor necrosis factor

* Statistically significant after Bonferroni correction

### Cytokine clusters and disease activity

**Fig. 3:**
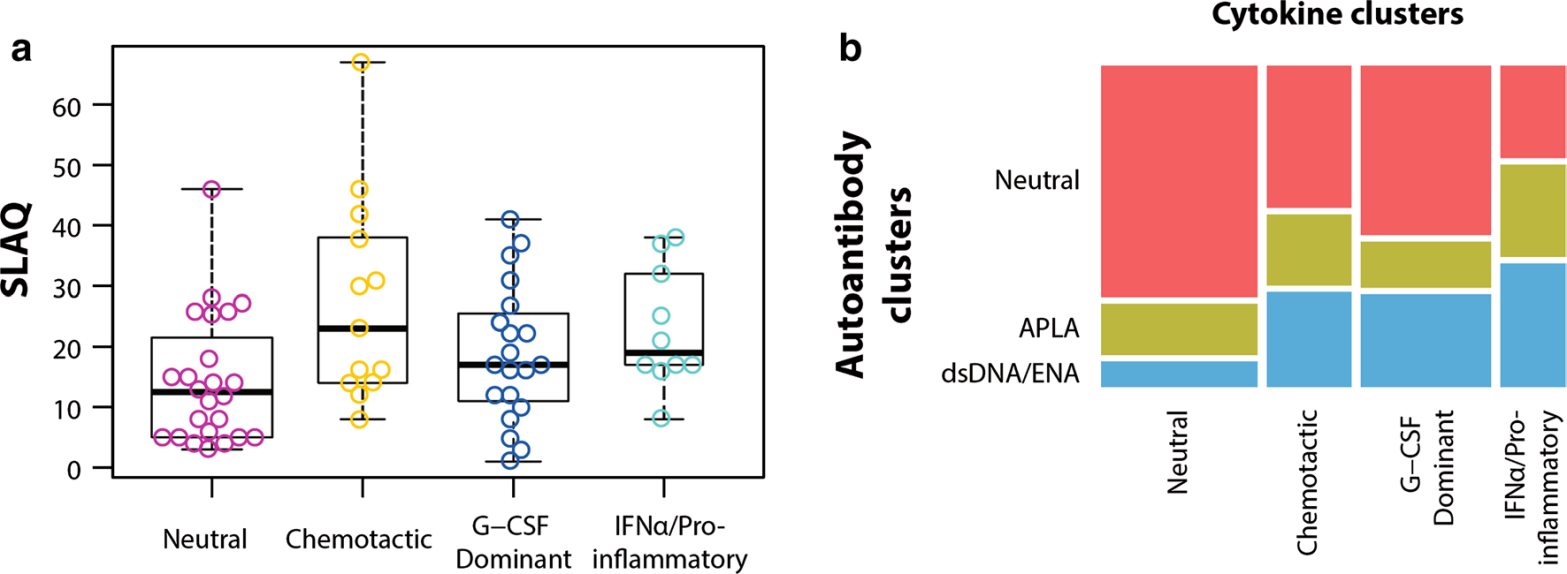
**a** Association between cytokine clusters and activity of disease (*p* = 0.022, by Kruskal–Wallis test). **b** Association between cytokine clusters and autoantibody clusters (*p* = 0.031 for the comparison between neutral and IFNα/Pro-inflammatory clusters, by Chi-square test)

There was a significant association between cytokine clusters and disease activity. (*p* = 0.022; Fig. 3a,). The distribution of autoantibody clusters differed between neutral cytokine and IFNα/Pro-inflammatory clusters (*p* = 0.031; Fig. 3b). Tables 6 and 7 show the distribution of cytokines by autoantibody clusters, and autoantibodies by cytokine clusters respectively.

**Table 6:** Distribution of cytokines by autoantibody clusters

Cytokine	Neutral (n = 38)	APLA-dominant (n = 13)	dsDNA/ENA-dominant (n = 16)	p-value
IL-2	0 (0)	2 (15)	0 (0)	0.013
IL-4	2 (5)	1 (8)	1 (6)	0.948
IL-5	0 (0)	4 (31)	2 (12)	0.003*
IL-6	6 (16)	5 (38)	4 (25)	0.229
IL-9	1 (3)	1 (8)	0 (0)	0.471
IL-10	2 (5)	7 (54)	5 (31)	0.0005*
IL-17A	6 (16)	3 (23)	2 (12)	0.737
TNFα	2 (5)	3 (23)	4 (25)	0.079
G-CSF	14 (37)	7 (54)	10 (62)	0.186
IFNα	7 (18)	4 (31)	6 (37)	0.299
IFNγ	3 (8)	1 (8)	0 (0)	0.512
IL-13	0 (0)	1 (8)	0 (0)	0.121
IL-1β	3 (8)	2 (15)	1 (6)	0.652
IL-8	7 (18)	4 (31)	5 (31)	0.486
IL-12/23p40	18 (47)	8 (61)	9 (56)	0.632

Data correspond to number of patients (%)

*IL* interleukin, *G-CSF* granulocyte colony-stimulating factor, *IFN* interferon, *TNF* tumor necrosis factor

* Statistically significant after Bonferroni correction

**Table 7:** Distribution of autoantibodies by cytokine clusters

Autoantibody	Neutral (n = 24)	Chemotactic (n = 13)	G-CSF (n = 20)	IFNα/Pro-inflammatory (n = 10)	p-value
RF	8 (33)	3 (23)	8 (40)	5 (50)	0.570
CCP3	1 (4)	0 (0)	0 (0)	0 (0)	0.610
TPOAb	2 (8)	1 (8)	4 (20)	0 (0)	0.342
TgAb	1 (4)	1 (8)	2 (10)	1 (10)	0.882
dsDNA	7 (29)	7 (54)	11 (55)	7 (70)	0.115
ACA-IgM	6 (25)	1 (8)	1 (5)	3 (30)	0.154
ACA-IgG	4 (17)	4 (31)	2 (10)	2 (20)	0.499
β2GP1-IgM	2 (8)	4 (31)	0 (0)	1 (10)	0.042
β2GP1-IgG	1 (4)	2 (15)	1 (5)	2 (20)	0.359
ANAs	18 (75)	12 (92)	19 (95)	8 (80)	0.239
RNP	4 (17)	7 (54)	10 (50)	4 (40)	0.062
Ro	9 (37)	4 (31)	7 (35)	6 (60)	0.496
La	1 (4)	1 (8)	2 (10)	1 (10)	0.882
Sm	1 (4)	3 (23)	5 (25)	3 (30)	0.172

Data correspond to number of patients (%)

*RF* rheumatoid factor, *CCP3* anti-cyclic citrullinated peptide third-generation, *TPOAb* anti-thyroperoxidase antibody, *TgAb* anti-thyroglobulin antibody, *dsDNA* anti-double stranded DNA antibodies, *ACA* anticardiolipin antibody, *β2GP1* β2 glycoprotein-1, *ANAs* antinuclear antibodies

### Cytokine and antibody clusters

**Fig. 4:**
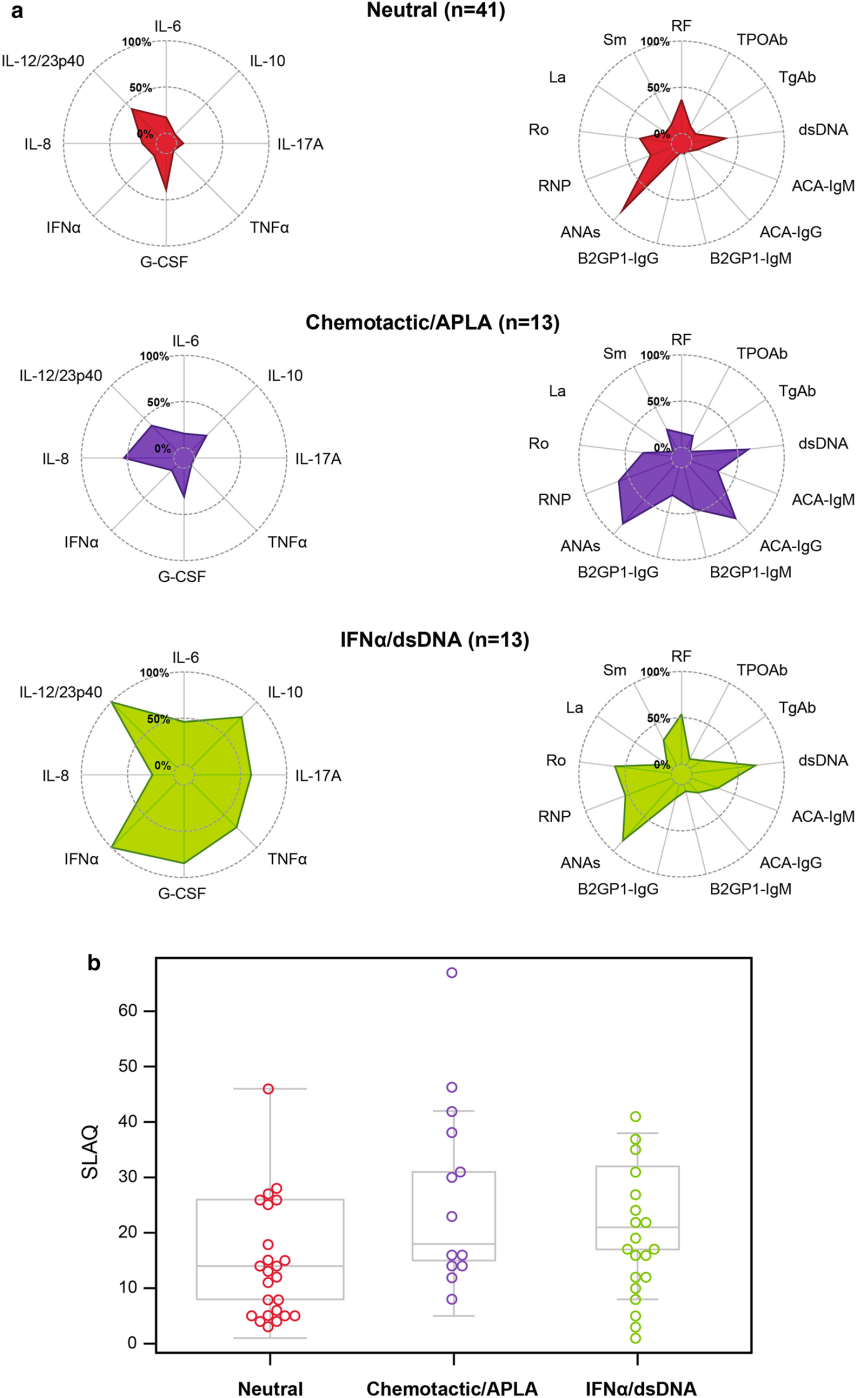
Integrative analysis. **a** Clusters of cytokines and autoantibodies. **b** Association between clusters and activity of disease (*p* = 0.036)

Finally, when all biomarkers were included (i.e., cytokines and autoantibodies), three clusters were found (Table 8): (1) neutral, (2) chemotactic/APLA, and (3) IFNα/dsDNA (Fig. 4a), which, in turn, evinced an association with SLE activity (p = 0.036; Fig. 4b). Differences among clusters with respect to clinical manifestations were not observed (Table 8).

**Table 8:** Distribution of autoantibodies and cytokines in integrative clusters

Biomarker	Neutral (n = 41)	Chemotactic/APLA (n = 13)	IFNα/dsDNA (n = 13)	p-value
Autoantibodies				
RF	15 (37)	2 (15)	7 (54)	0.121
CCP3	1 (2)	0 (0)	0 (0)	0.724
TPOAb	4 (10)	2 (15)	1 (8)	0.792
TgAb	3 (7)	0 (0)	2 (15)	0.327
dsDNA	15 (37)	8 (61)	9 (69)	0.065
ACA-IgM	3 (7)	4 (31)	4 (31)	0.041
ACA-IgG	0 (0)	10 (77)	2 (15)	< 0.0001*
β2GP1IgM	0 (0)	6 (46)	1 (8)	< 0.0001*
β2GP1IgG	0 (0)	4 (31)	2 (15)	0.0021
ANAs	35 (85)	11 (85)	11 (85)	0.996
RNP	10 (24)	8 (61)	7 (54)	0.021
Ro	14 (34)	4 (31)	8 (61)	0.168
La	4 (10)	0 (0)	1 (8)	0.506
Sm	5 (12)	3 (23)	4 (31)	0.271
Cytokines				
IL-2	0 (0)	1 (8)	1 (8)	0.196
IL-4	0 (0)	0 (0)	4 (31)	0.0001*
IL-5	0 (0)	1 (8)	5 (38)	0.0001*
IL-6	7 (17)	2 (15)	6 (46)	0.072
IL-9	1 (2)	0 (0)	1 (8)	0.487
IL-10	1 (2)	3 (23)	10 (77)	< 0.0001*
IL-17A	3 (7)	0 (0)	8 (61)	< 0.0001*
TNFα	0 (0)	0 (0)	9 (69)	< 0.0001*
G-CSF	16 (39)	4 (31)	11 (85)	0.007
IFNα	3 (7)	1 (8)	13 (100)	< 0.0001*
IFNγ	2 (5)	0 (0)	2 (15)	0.227
IL-13	0 (0)	0 (0)	1 (8)	0.121
IL-1β	1 (2)	0 (0)	5 (38)	0.0001*
IL-8	6 (15)	7 (54)	3 (23)	0.015
IL-12/23p40	17 (41)	5 (38)	13 (100)	0.0006*
1997 ACR criteria				
Positive ANAs	34 (83)	11 (85)	13 (100)	0.282
Immunologic criteria	32 (78)	10 (77)	12 (92)	0.491
Hematologic criteria	28 (68)	13 (100)	7 (54)	0.024
Non-erosive arthritis	28 (68)	7 (54)	12 (92)	0.092
Photosensitivity	26 (63)	8 (61)	7 (54)	0.826
Malar rash	20 (49)	7 (54)	6 (46)	0.921
Renal criteria^a^	14 (34)	6 (46)	5 (38)	0.734
Oral ulcers	14 (34)	5 (38)	4 (31)	0.917
Serositis	10 (24)	5 (38)	4 (31)	0.604
Neurologic criteria	4 (10)	4 (31)	4 (31)	0.091
Discoid rash	3 (7)	3 (2)	1 (8)	0.252

Data correspond to number of patients (%)

*RF* rheumatoid factor, *CCP3* anti-cyclic citrullinated peptide third-generation, *TPOAb* anti-thyroperoxidase antibody, *TgAb* anti-thyroglobulin antibody, *dsDNA* anti-double stranded DNA antibodies, *ACA* anticardiolipin antibody, *β2GP1* β2 glycoprotein-1, *ANAs* antinuclear antibodies, *IL* interleukin, *G-CSF* granulocyte colony-stimulating factor, *IFN* interferon, *TNF* tumor necrosis factor, *ACR* American College of Rheumatology

* Statistically significant after Bonferroni correction

^a^Renal criteria was defined as active urinary sediment, or proteinuria > 500 mg/24 h or positive renal biopsy [23, 24]

## Discussion

The results indicate the presence of three cytokine-autoantibody driven subphenotypes in SLE. First, three autoantibody clusters were identified, namely (1) neutral, (2) APLA-dominant and (3) anti-dsDNA/ENA-dominant. Cluster analyses in SLE patients have been done previously and tended to show similar results even among different populations and clustering methods (Additional file 2: Table S1). The first report, by Tápanes et al. [16], assessed the relationship between renal outcomes and anti-ENA clusters and proposed 4 clusters based on ENA positivity (no ENA, Ro/La, Sm/RNP, all positive). In the current study, the neutral autoantibody cluster showed a low frequency of autoantibodies, where ANAs stood out as the most abundant. Along the same line, Artim-Esen et al. [8] described a cluster that showed only ANA positivity, a rather unspecific autoantibody that could be similar to our neutral cluster. Furthermore, several cohorts have shown a particular cluster characterized by anti-dsDNA solely [8, 9, 12, 15]. Although this antibody presents with high frequency in SLE patients [37] it does not allow clusters to be differentiated in other cohorts [7, 11].

A second autoantibody cluster in the current study was dominated by APLA. These autoantibodies were not included in cluster studies until recently. Artim-Esen et al. [8] and To et al. [38] found an APLA dominant cluster which was similar to our results.

A cluster characterized by the presence of anti Sm/RNP antibodies has been consistently reported [7, 10, 14, 15], and in some reports, it has been associated with anti-dsDNA, thus yielding a Sm/RNP/dsDNA cluster [8, 12]. Likewise, a Ro/La cluster has also been reported [9, 10, 15], and in some reports, it has been associated with anti-dsDNA [8, 11, 14]. These findings are supported by the cross-reactivity and similarity of anti-Sm and anti-RNP [39], and the induction of anti-Ro and anti-La by common ribonucleoproteins [40]. It is noteworthy that some authors have found one cluster with positivity for 3 or 4 ENA (with or without anti-dsDNA) [7, 11, 12]. This evidence is similar to our Cluster 3 in which a predominance of anti-dsDNA/ENA was observed. Anti-Sm and anti-La antibodies were virtually absent in neutral and APLA autoantibody clusters (Fig. 1).

Autoantibody clusters did not show an association with disease activity [12, 14]. This could be due to the measurement method, in which a well-known PRO questionnaire (i.e., SLAQ) [41] was used in contrast to physician-based indexes (i.e., SLEDAI) used in other cohorts. Nevertheless, there is no serologic test that reliably measures disease activity in SLE [42].

Second, four cytokine clusters were obtained, namely (1) neutral, (2) chemotactic, (3) G-CSF dominant, and 4) IFNα/Pro-inflammatory. To our knowledge, this is the first report on a serum cytokine cluster analysis in patients with SLE. The composition of the third and fourth cytokine clusters were validated by an external bioinformatic analysis which confirmed biological relationships among cytokines (Additional file 3).

The neutral cytokine cluster displayed a low frequency of cytokines that was below 25% (Fig. 2). The chemotactic cytokine cluster showed a marked expression of IL-8 followed by lower frequencies of IL-12/23p40, IL-6, and G-CSF. IL-8 is a chemotactic cytokine, particularly involved in recruitment of neutrophils, which induces shape transformation, the ‘respiratory burst’, and the release of granule contents [43]. Increased levels have been seen in SLE patients and they appear to be influenced by anti-dsDNA (Additional file 4: Table S2). High levels of IL-6 have also been found in SLE [44, 45]. Both IL-6 and IL-8 have been shown to be up-regulated by endothelial cells treated with IgG APLA in vitro [46]. IL-8 has been associated with pregnancy morbidity in patients with SLE [35]. The anti-dsDNA antibody up-regulates IL-8 gene expression and elicits activation-induced cell death of human polymorphonuclear neutrophils [47], and the release of IL-8 [48].

The third cytokine cluster was named G-CSF dominant, since a particularly high frequency of G-CSF was seen although IL-12/23p40 was rather frequent. G-CSF is an essential growth factor for the differentiation of hematopoietic stem cells into granulocytes, particularly neutrophils. Synthetic G-CSF preparations (e.g., filgrastim, pegfilgrastim, lenograstim) are available to treat neutropenia [49]. Data is scarce regarding G-CSF in SLE (Additional file 4: Table S2). Furthermore, IL-12 and IL-23 are mainly pro-inflammatory cytokines that share a common structural unit and receptors. IL-12 consists of two subunits: p35 and p40, whereas IL-23 is comprised of subunits p19 and p40. The two share the p40 subunit, which interacts with the same membrane receptor [50]. Although available assays for p19, p35, and p70 (which includes subunits IL-12 p35 and p40) exist, we measured IL-12/23p40. IL-12 is pivotal for Th1 differentiation [51] and has been found to be higher in SLE patients [52]; its implication for physiopathology remains under investigation (Additional file 4: Table S2). In addition, IL-23 plays a role in the development of Th17 cells, and promotes IL-17 secretion [53]; clinical trials with anti-IL-12/23 are underway [50].

The last cytokine cluster revealed high levels of diverse cytokines, including G-CSF, IL-12/23p40, IL-17A, and IL-10. Nonetheless, IFNα and TNFα were the most frequent cytokines. Thus, the cluster was named IFNα/Pro-inflammatory. IFNα belongs to the Type I IFN family and is mainly secreted by plasmacytoid dendritic cells (pDC). Type I IFNs promote autoimmunity due to the activation of B-cell responses, maturation of monocytes into DC, and NETosis promotion [4, 54]. One of the SLE hallmarks is its IFN I signature, which is dysregulated when compared to healthy controls (Additional file 4: Table S2). Recent clinical trials in SLE with a Type I IFN blockade (i.e., sifalimumab, anifrolumab) have shown promising results [55, 56].

TNFα is a pro-inflammatory cytokine secreted by monocytes, macrophages, T cells, neutrophils, and mast cells. It promotes lymphocyte recruitment and inflammatory responses. However, it becomes immunosuppressive with chronic exposure [57]. Increased levels, which correlated with disease activity, have been found in SLE patients (Additional file 4: Table S2). A negative feedback loop between Type I IFN and TNFα has been suggested: when Type I IFN prevails, SLE may occur. TNF inhibits the development of pDCs and their production of Type I IFN [58]. IL-17A belongs to the IL-17 family. It is largely produced by Th17 cells although it is secreted by several immune cells [59]. IL-17A has been widely studied in autoimmunity and is thought to play a pivotal role in SLE physiopathology (Additional file 4: Table S2). IL-10 is an immunomodulatory cytokine secreted by several cell populations although it exerts an essential role in B cell processes. Thus, it may promote hyperactivity of the B-cell compartment, thus leading to increased autoantibody production. Due to its dual function (i.e., B-cell stimulation/antibody production, and T-cell inflammatory response reduction), its role in SLE is not fully understood (Additional file 4: Table S2). IL-6 is a multifunctional cytokine secreted by several cells of both the innate and adaptive immune systems as well as by non-immune cells such as fibroblasts [60]. Increased serum levels are found in SLE and appear to be associated with joint involvement [44, 61, 62], and disease activity [52]. A few monoclonal antibodies (e.g., tocilizumab, sarilumab) are current treatment options for different ADs [4]. Nevertheless, randomized clinical trials for SLE are lacking. A summary of the role of IL-6 in SLE is shown in Additional file 4: Table S2.

Some cytokines exhibited a low prevalence (< 5%) (i.e., IL-1β, IL-2, IL-4, IL-5, IL-9, IL-13, IFNγ) in most patients, and thus did not account for any cluster. IL-1β is secreted mainly by innate immune cells. High serum levels are uncommon among SLE patients and appear to lack an association with SLE pathogenesis [63] (Additional file 4: Table S2).

IL-2 is predominantly produced by Tregs. Deficiency in IL-2 secretion is involved in the pathogenesis of SLE through the impairment of Treg growth and survival [64]. Recent evidence has shown that IL-2 secretion is impaired by high levels of IL-23 and IL-23R [65]. This may explain the absence of IL-2 in our clusters due to the high frequency of IL-12/23p40 in patients (Additional file 4: Table S2). IL-4 is secreted by several immune cells, particularly basophils. Since it stimulates B-cells, it may be involved in antibody production and SLE pathophysiology (Additional file 4: Table S2). IL-9 is a pleiotropic cytokine, produced by an ample variety of immune cells including mast cells, NKT cells, Th2, Th17, Treg, and the recently described Th9 [66]. It is considered a proliferative cytokine, which can induce the production of IL-6, mainly by mast cells. It enhances B-cell production of IL-4, IgE, and IgG1 and promotes isotype switching. In contrast, IL-9 secreted by Treg induces tolerance [66]. Some data on the implications of IL-9 for SLE have been described (Additional file 4: Table S2). IFNγ is mainly produced by T and NK cells. It is important for T cell differentiation and B-cell isotype switching [67]. Its role in SLE is described in Additional file 4: Table S2. Data regarding IL-5 and IL-13 in SLE patients is scarce. IL-5 is pivotal for eosinophil differentiation [68], whereas IL-13 is an immunomodulatory cytokine secreted by Th2 CD4 T-cells (Additional file 4: Table S2).

Regarding the interaction between autoantibodies and cytokines, the absence of autoantibodies was associated with a low frequency of cytokines (Table 6). Disease activity, in turn, was lower in the neutral cytokine cluster (Fig. 3a). The enhancement of antibody production and activation of autoreactive B cells may be favored by a Th2 environment [51, 69]. Some inflammatory cytokines (e.g., TNFα, G-CSF) were less common in the neutral autoantibody cluster although statistical significance was not reached (Table 6). IL-10 was significantly lower in the neutral autoantibody cluster than in the APLA-dominant and dsDNA/ENA dominant clusters (Table 6). Abnormally increased IL-10 synthesis seems contributing to the spontaneous hyperactivity of the B cell compartment, so that it can directly result in autoantibody production by committed plasma cells, circulating immune complexes formation, and eventually in tissue and organ damage [70]. IL-5 was absent in the neutral autoantibody cluster, which may be in line with the reported worse renal outcomes associated with elevated IL-5 urine concentration [68]. Additionally, anti-dsDNA antibodies were less frequent within the neutral cytokine cluster (Table 4), which highlights the involvement of these autoantibodies in SLE pathophysiology. The IFNα/Pro-Inflammatory cluster revealed a significant low frequency of the neutral autoantibody cluster (Fig. 3b). This finding is in line with the capability of IFNs and IL-17 to induce antibody secretion [54, 71]. These results support the fact that SLE disease activity is mediated by cytokine secretion [2] and the potential role of autoantibodies in the enhancement of cytokine production [48, 72].

Our last analysis, which was integrative and in which all the biomarkers were included, showed three clusters which reinforced the results (Fig. 4a). The G-CSF cytokine cluster observed in the second analysis was distributed throughout the three integrative clusters. In addition, these three clusters were associated with the disease activity (Fig. 4b).

Our study supports the importance of individualized treatment of patients since both autoantibody and cytokine clusters were established in a cohort of SLE patients that also showed interaction and association with disease activity. The identification of SLE subphenotypes has been suggested previously [73] and is pivotal for the implementation of personalized medicine [74]. Our results depict the existence of different subphenotypes based on both diverse disease-specific and non-specific autoantibodies and on easy-access molecules such as cytokines. A comprehensive assessment of multiple biomarkers, which is feasible with multiplex assay technologies [27], should offer the possibility of a novel taxonomy for SLE and the implementation of targeted therapies based on cytokine patterns (i.e., proof-of-concept studies).

Systems biology approaches have been applied to rheumatic diseases, in order to find novel biomarkers and therapeutic strategies. These efforts have focused on gene-level interactions and their relationship with clinical manifestations. For instance, Chiche et al. [75] found three individual IFN modules through transcriptional repertoire analysis using microarray technology, which showed an association with some clinical variables. Likewise, Bancherau et al. [6] described seven discrete groups of SLE patients based on their gene expression patterns and clinical disease severity. Reclassification of SLE patients based on the results of ‘*omics*’ studies has been proposed [76].

The possible shortcomings of our study must be acknowledged. The main objective was to evaluate simultaneously the relationship among cytokines, autoantibodies, and the disease activity at one point in time. Therefore, the lack of association between clusters and cumulative clinical characteristics was expected. Likewise, the effects of treatment on the modulation of cytokine/autoantibody levels were not taken into account. The main considered outcome was the activity of the disease. However, the results of this exploratory study should stimulate further longitudinal designs using larger groups of patients to fully describe these subtle complexities. Regarding the cytokine measurement method, previous reports on RA patients’ sera showed that the correlation of CBA assays with ELISA-based methods for cytokine detection is moderately-high although a lower concentration for some cytokines (i.e., IL-2, TNFα, IL-10) may be detected [77]. Furthermore, CBA is not affected by the presence of RF in contrast to other multiplex technologies (e.g., Luminex) and allows the assessment of multiple biomarkers using relatively small sample volumes [77]. We are aware of the reported low positivity of IFNα levels in serum, which is rarely detectable by ELISA or bioassays, and the suggested assessment of gene expression monitoring [78]. However, our results yielded appropriate IFNα assessment by CBA and even the identification of an IFNα-related cluster. Measurements of IFNγ using CBA appear to be proper which supports a low concentration in our patients [77]. Although other techniques are used in research to measure cytokines, CBA is implemented in many clinical and regular laboratories. This is, therefore, a cost-effective and practical method. Another potential drawback of our study could be the method of disease activity quantification, SLAQ, a well-known PRO questionnaire [41], which was used under a non-structured validation. Since SLE may exhibit a disconnection between disease activity and patient perceived well-being, PROs may help empower patients on disease management. Numerous regulatory agencies encourage the use of PROs in clinical trials [79]. Note that our results showed an association between SLAQ scores and cytokine clusters. This fact is particularly interesting given that SLAQ does not include autoantibodies (e.g., anti-dsDNA) in its criteria in contrast to clinical indexes (e.g., SLEDAI). Thus, disease activity could be biased by anti-dsDNA. Instead, as shown herein, disease activity as portray by SLAQ may be due to others biomarkers such as cytokines. Another potential limitation of the present study is that the observed results may be due to chance alone or the moderate sample size. However, such a possibility would be unlikely given the highly significant results seen as well as their consistent direction and magnitude within the different analyses.

## Conclusions

Systemic lupus erythematosus is a heterogeneous systemic AD with profound cytokine abnormalities. Multiple disease-specific and non-specific biomarkers are present in SLE patients. Clustering methods allow the identification of association among these markers and yield different subphenotypes. Additional systems medicine approaches are warranted in order to reveal the strength of these interactions, which should assist in the implementation of personalized medicine.

## Additional files

**Additional file 1.** SLAQ_SpanishValid. Systemic lupus activity questionnaire (SLAQ) – Spanish linguistic validation. Spanish linguistic validation of SLAQ as described in “Methods” section.

**Additional file 2.** SLE autoantibody clusters_Summary. SLE autoantibody clusters in the literature – Summary table. Summary table of the available autoantibody clusters in the literature regarding SLE patients. Author, country, clustering method, sample size, and found clusters are included.

**Additional file 3.** SLE clusters_Bioinformatics analysis. Bioinformatic analysis for G-CSF (CSF3) – Dominant cluster and IFNα/Pro-inflammatory cluster. Bioinformatic analysis for G-CSF (CSF3) – Dominant cluster and IFNα/Pro-inflammatory cluster showing known interactions between cytokines, based on ‘STRING: functional protein association networks’ (https://string-db.org/).

**Additional file 4.** Summary of cytokine implicated in SLE. Summary of cytokines implicated in SLE—Summary table. Review of literature on the main cytokines implicated in SLE.

## Abbreviations

SLE: systemic lupus erythematosus
AD: autoimmune disease
RF: rheumatoid factor
CCP: cyclic citrullinated peptide
APLA: antiphospholipid antibodies
APS: antiphospholipid syndrome
TPOAb: anti-thyroid peroxidase antibodies
TgAb: anti-thyroglobulin antibodies
CREA: Center for Autoimmune Diseases Research
ACR: America College of Rheumatology
SLAQ: Systemic Lupus Activity Questionnaire
CCP3: anti-CCP third-generation
ACA: anti-cardiolipin antibodies
2GP1: anti-2 glycoprotein-1
dsDNA: double-stranded DNA
ELISA: enzyme-linked immunosorbent assay
ANAs: antinuclear antibodies
IL: interleukin
G-CSF: granulocyte colony-stimulating factor
IFN: interferon
TNF: tumor necrosis factor
CBA: cytometric bead array
ENA: extractable nuclear antigen antibodies
PRO: patient-reported outcome
SLEDAI: Systemic Lupus Erythematosus Disease Activity Index
DC: dendritic cell
pDC: plasmacytoid dendritic cell
NETosis: neutrophil extracellular traps
NK: natural killer
Ab: antibody
