# Supplementary material for: Cytokine and autoantibody clusters interaction in systemic lupus erythematosus
Source: J Transl Med. 2017 Nov 25;15:239. doi: 10.1186/s12967-017-1345-y (PMC5702157; doi:10.1186/s12967-017-1345-y)
Supplement: Supplementary file 1 — Additional file 1. SLAQ_SpanishValid. Systemic lupus activity questionnaire (SLAQ) – Spanish linguistic validation. Spanish linguistic validation of SLAQ as described in “Methods” section. [file 12967_2017_1345_MOESM1_ESM.docx]

**SYSTEMIC LUPUS ACTIVITY QUESTIONNAIRE (SLAQ)**

Por favor responda y marque con una **X** según corresponda:

1. En los últimos 3 meses, ¿ha tenido una exacerbación de lupus? (una exacerbación de lupus es cuando su lupus se pone peor). ¿Cuál de las siguientes describe mejor su respuesta? (por favor **marque con una x**)

No, ninguna exacerbación.

Si, exacerbación leve.

Si, exacerbación moderada.

Si, exacerbación severa.

1. Por favor, revise el siguiente listado de síntomas de lupus. **EN LOS ULTIMOS 3 MESES**, ¿Qué tan mal ha estado cada uno de los síntomas? Por favor marque con una x para cada síntoma.

|  | **NO HAY PROBLEMA** | **LEVE** | **MODERADO** | **SEVERO** |
| --- | --- | --- | --- | --- |
| Pérdida de peso no intencionada. |  |  |  |  |
| Fatiga. |  |  |  |  |
| Fiebre (más de 38.5 grados) medida con termómetro. |  |  |  |  |
| Llagas en la boca o la nariz. |  |  |  |  |
| Brote en la piel de las mejillas (forma de mariposa) |  |  |  |  |
| Otros brotes en la piel ¿Dónde? |  |  |  |  |
| Manchas de color morado o azul oscuro que podía sentir sobre su piel |  |  |  |  |
| Brotes en la piel o sensación de malestar después de salir al sol. |  |  |  |  |
| Parches de calvicie en la cabeza o mechones de cabello sobre la almohada. |  |  |  |  |
| Ganglios inflamados (nódulos) en el cuello. |  |  |  |  |
| Dificultad para respirar. |  |  |  |  |
| Dolor en el pecho con una respiración profunda. |  |  |  |  |
| Dedos de las manos o pies se ponen blancos o muy pálidos con el frío (Raynaud). |  |  |  |  |
| Dolor de estómago o de vientre. |  |  |  |  |
| Adormecimiento u hormigueo persistente en sus brazos o piernas. |  |  |  |  |
| Convulsiones. |  |  |  |  |
| Derrame cerebral. |  |  |  |  |
| Pérdida de memoria. |  |  |  |  |
| Sentimiento de depresión. |  |  |  |  |
| Dolores de cabeza inusuales. |  |  |  |  |
| Dolor muscular. |  |  |  |  |
| Dolor o rigidez de las articulaciones. |  |  |  |  |
| Inflamación en las articulaciones |  |  |  |  |

1. Por favor, califique la actividad de su lupus **DURANTE LOS ULTIMOS 3 MESES** en la escala mostrada a continuación, donde **0 es sin actividad** y **10 es la mayor actividad** (marque con una x).

**Máxima actividad**

**Ausencia de actividad**

| 0 | 1 | 2 | 3 | 4 | 5 | 6 | 7 | 8 | 9 | 10 |
| --- | --- | --- | --- | --- | --- | --- | --- | --- | --- | --- |
