# Supplementary material for: Cytokine and autoantibody clusters interaction in systemic lupus erythematosus
Source: J Transl Med. 2017 Nov 25;15:239. doi: 10.1186/s12967-017-1345-y (PMC5702157; doi:10.1186/s12967-017-1345-y)
Supplement: Supplementary file 2 — Additional file 2. SLE autoantibody clusters_Summary. SLE autoantibody clusters in the literature – Summary table. Summary table of the available autoantibody clusters in the literature regarding SLE patients. Author, country, clustering method, sample size, and found clusters are included. [file 12967_2017_1345_MOESM2_ESM.docx]

| **Author/**  **Country** | **n** | **Clustering Method** | **Number of clusters** | **ANAs** | **APLAs** | **ENA/dsDNA** | **ENA** | **dsDNA** | **ENA/dsDNA/**  **APLA** | **Ribosomal-P** | **Histone** |
| --- | --- | --- | --- | --- | --- | --- | --- | --- | --- | --- | --- |
| Current/  Colombia | 67 | Mixed-cluster | 3 | X | X | X |  |  |  |  |  |
| Tápanes/  Venezuela[1] | 91 | HCA^*^ | 4^1^ |  |  | X |  | X |  |  |  |
| Artim-Esen/  Turkey[2] | 852 | 2 steps cluster analysis  (Hierarchical) | 5^2^ | X | X | X |  | X |  |  |  |
| Li/Hong Kong[3] | 1928 | Non-hierarchical  K-means | 3^3^ |  |  | X |  | X | X |  |  |
| Jurencak/Canada[4] | 156 | Mixed-cluster | 3^4^ |  |  | X |  | X |  |  |  |
| Hoffman/Holland-Belgium-Slovakia-UK[5] | 235 | HCA^*^ | 5^5^ |  |  | X |  | X |  | X | X |
| Tan/  Singapur[6] | 64 | Non-hierarchical K-means | 3^6^ |  |  | X |  |  | X |  |  |
| Tang/  China[7] | 917 | Non-hierarchical K-means | 3^7^ | X |  | X |  |  |  |  |  |
| Ching/  China[8] | 205 | Ad-Hoc | 3^8^ | X |  | X |  |  |  |  |  |
| To/USA[9] | 1357 | Non-hierarchical K-means | 3^9^ |  | X | X | X |  |  |  |  |
| Park/Korea  (EULAR 2017 SAT0275)[10] | 339 | Non-hierarchical  K-means | 3^10^ |  |  | X | X |  |  |  |  |

**Table S1.** Cluster analysis in SLE patients available in the literature.

^*^Hierarchical cluster analysis.

^1^ 1) no-ENA, 2) Ro/La, 3) Sm/RNP, 4) All-ENA. ^2^ 1) ANAs solely, 2) dsDNA solely, 3) Sm/RNP, 4) ACA IgG-IgM/LAC, 5) Ro/La. ^3^ 1) dsDNA, 2) Sm/RNP/APLA, 3) Ro/La. ^4^ 1) dsDNA, 2) dsDNA/anti-chromatin/anti-ribosomal P/U1-RNP/Sm/Ro/La, 3) dsDNA/RNP/Sm. ^5^ 1)SmB/SmD/RNP-A/RNP-C/RNP-70k, 2) Ro52/Ro60/La, 3) anti-ribosomal P, 4) anti-histones, 5) dsDNA. ^6^ 1) dsDNA/Sm/U1-RNP/Ro/La/ACA IgM-IgG, 2) Sm/U1-RNP, 3) dsDNA/Ro. ^7^ 1) Ro/Sm/RNP, 2) Ro, 3) No-ENA (ANAs and low frequency of RNP and La). ^8^ 1) Sm/RNP, 2) Ro/La. ^9^ 1) Sm/RNP, 2) dsDNA/Ro/La, 3) dsDNA/ACA IgM-IgG/LAC. ^10^ 1) dsDNA/Ro, 2) RNP, 3) RNP/Ro/La.

1. Tápanes FJ, Vásquez M, Ramírez R, Matheus C, Rodríguez MA, Bianco N. Cluster analysis of antinuclear autoantibodies in the prognosis of SLE nephropathy: are anti-extractable nuclear antibodies protective? Lupus. 2000;9:437–44.

2. Artim-Esen B, Çene E, Şahinkaya Y, Ertan S, Pehlivan Ö, Kamali S, et al. Cluster analysis of autoantibodies in 852 patients with systemic lupus erythematosus from a single center. J. Rheumatol. 2014;41:1304–10.

3. Li PH, Wong WHS, Lee TL, Lau CS, Chan TM, Leung AMH, et al. Relationship between autoantibody clustering and clinical subsets in SLE: cluster and association analyses in Hong Kong Chinese. Rheumatology. 2013;52:337–45.

4. Jurencák R, Fritzler M, Tyrrell P, Hiraki L, Benseler S, Silverman E. Autoantibodies in pediatric systemic lupus erythematosus: ethnic grouping, cluster analysis, and clinical correlations. J. Rheumatol. 2009;36:416–21.

5. Hoffman IEA, Peene I, Meheus L, Huizinga TWJ, Cebecauer L, Isenberg D, et al. Specific antinuclear antibodies are associated with clinical features in systemic lupus erythematosus. Ann. Rheum. Dis. 2004;63:1155–8.

6. Tan JHT, Hoh SF, Win MTM, Chan YH, Das L, Arkachaisri T. Childhood-onset systemic lupus erythematosus in Singapore: clinical phenotypes, disease activity, damage, and autoantibody profiles. Lupus. 2015;24:998–1005.

7. Tang X, Huang Y, Deng W, Tang L, Weng W, Zhang X. Clinical and serologic correlations and autoantibody clusters in systemic lupus erythematosus: a retrospective review of 917 patients in South China. Medicine (Baltimore). 2010;89:62–7.

8. Ching KH, Burbelo PD, Tipton C, Wei C, Petri M, Sanz I, et al. Two major autoantibody clusters in systemic lupus erythematosus. PLoS One. 2012;7:e32001.

9. To CH, Petri M. Is antibody clustering predictive of clinical subsets and damage in systemic lupus erythematosus? Arthritis Rheum. 2005;52:4003–10.

10. Park DJ, Kang JH, Kim JE, Lee KE, Lee SS. Comparison of clinical and serological differences according to the autoantibody cluster in women with systemic lupus erythematosus: results from the Korean lupus network (KORNET) registry. Ann. Rheum. Dis. 2017;76 Suppl 2:SAT0275.
