# Supplementary material for: Cytokine and autoantibody clusters interaction in systemic lupus erythematosus
Source: J Transl Med. 2017 Nov 25;15:239. doi: 10.1186/s12967-017-1345-y (PMC5702157; doi:10.1186/s12967-017-1345-y)
Supplement: Supplementary file 3 — Additional file 3. SLE clusters_Bioinformatics analysis. Bioinformatic analysis for G-CSF (CSF3) – Dominant cluster and IFNα/Pro-inflammatory cluster. Bioinformatic analysis for G-CSF (CSF3) – Dominant cluster and IFNα/Pro-inflammatory cluster showing known interactions between cytokines, based on ‘STRING: functional protein association networks’ (https://string-db.org/). [file 12967_2017_1345_MOESM3_ESM.pptx]

## Slide 1
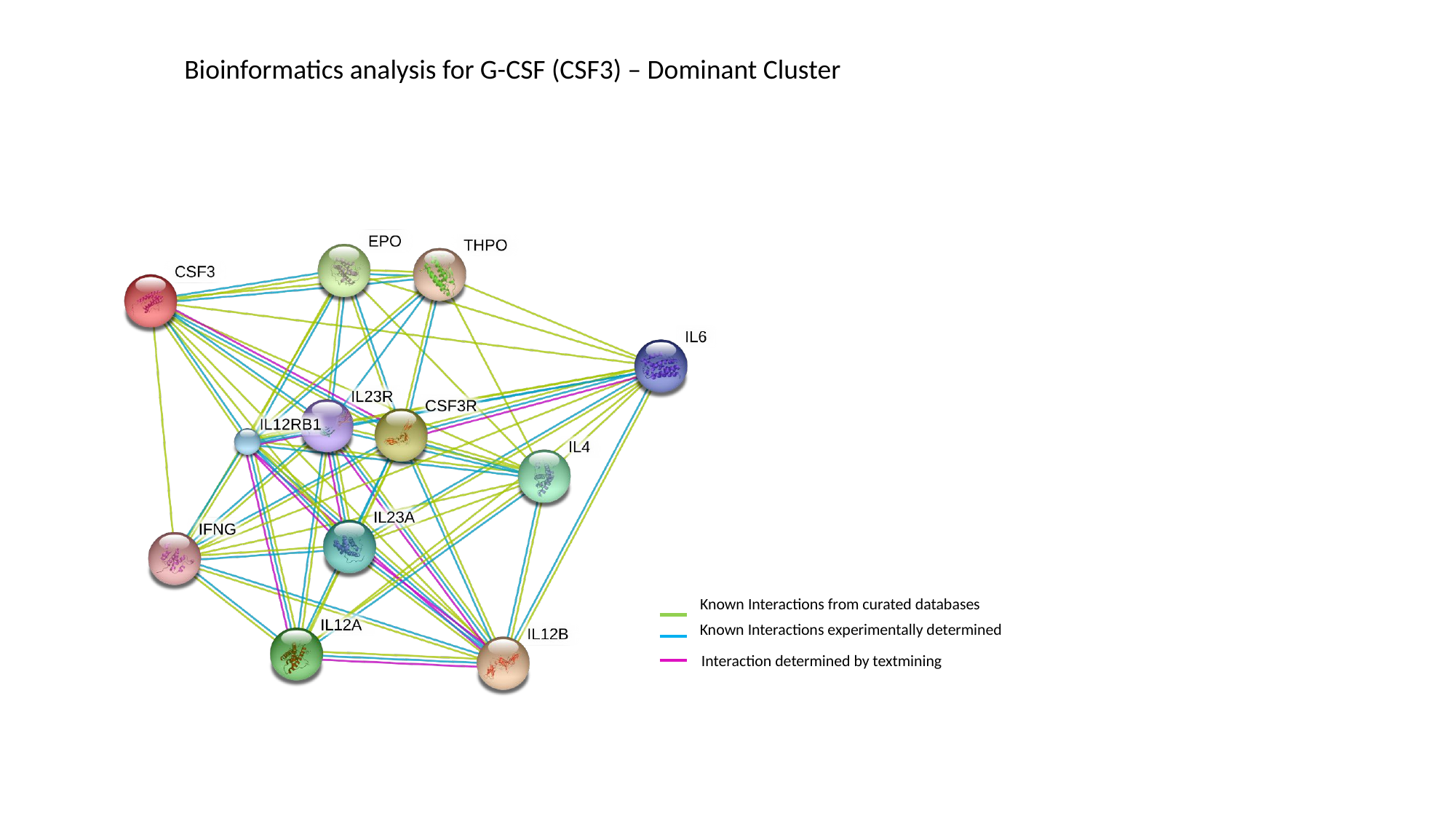

Bioinformatics analysis for G-CSF (CSF3) – Dominant Cluster
Known Interactions from curated databases
Known Interactions experimentally determined
Interaction determined by textmining

## Slide 2
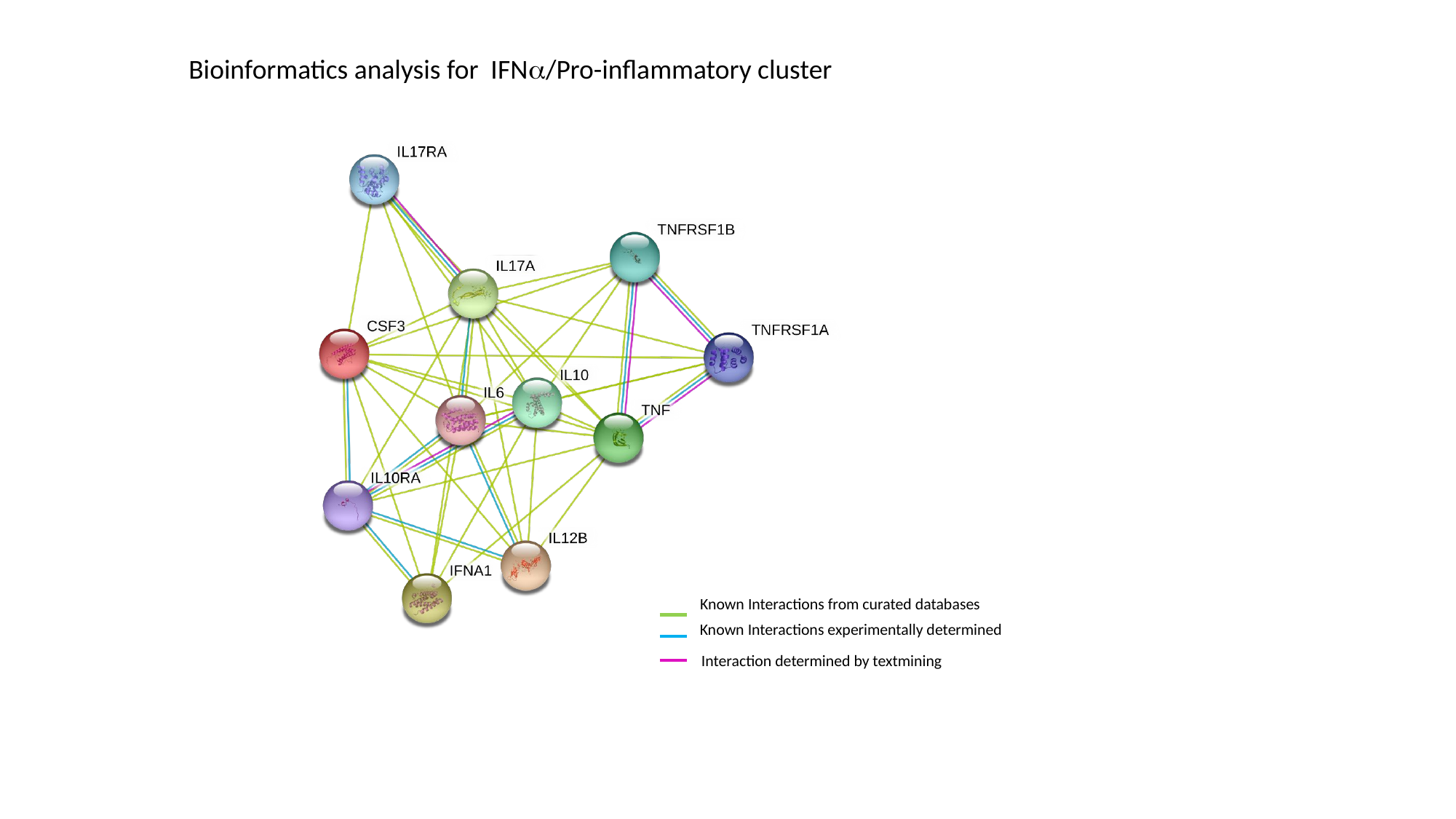

Bioinformatics analysis for IFNa/Pro-inflammatory cluster
Known Interactions from curated databases
Known Interactions experimentally determined
Interaction determined by textmining
