# Supplementary material for: Cytokine and autoantibody clusters interaction in systemic lupus erythematosus
Source: J Transl Med. 2017 Nov 25;15:239. doi: 10.1186/s12967-017-1345-y (PMC5702157; doi:10.1186/s12967-017-1345-y)
Supplement: Supplementary file 4 — Additional file 4. Summary of cytokine implicated in SLE. Summary of cytokines implicated in SLE—Summary table. Review of literature on the main cytokines implicated in SLE. [file 12967_2017_1345_MOESM4_ESM.docx]

**Additional file 4. Table S2.** Summary of cytokines evaluated and their implications in SLE

| **Cytokine** | **Implications in SLE** | **References** |
| --- | --- | --- |
| **IL-8** | Involved in renal injury. A polymorphism within 5’ flanking region of IL-8 gene, probably involved in IL-8 translational start. | [1] |
|  | Anti-dsDNA antibodies might induce IL-8 secretion in renal tubular epithelial cells increasing the inflammation and contributing to renal nephritis in mice. | [2,3] |
|  | Increased on serum and levels failed to recover after immunosuppressive treatment. Concentration in cerebrospinal fluid from patients with neuropsychiatric lupus is higher than in healthy donors. | [4–8] |
|  | Urinary levels are associated with lupus activity and lupus nephritis. | [9] |
|  | Induces neutrophil recruitment and extracellular traps formation (NETosis) increasing the risk of anti-nuclear autoantibodies production. | [10,11] |
| **IL-6** | Serum levels are associated with SLE activity and joint impairment. IL-6 blockade induces improvement in lupus arthritis. | [12–17] |
|  | Associated with SLE risk. Uncontrolled IL-6 secretion by B-cells may be restored after treatment on responsive patients. | [18] |
|  | IL-6 polymorphism is a risk for SLE. | [19] |
| **G-CSF** | High doses of G-CSF treatment on SLE-prone mice have shown a protective role against SLE, whereas low doses increase SLE severity. | [20] |
|  | In a mouse model, G-CSF promotes Treg cells proliferation inducing decrease of anti-dsDNA antibodies levels in sera and decreasing lupus nephritis risk. Also, G-CSF is associated with reduced mortality in mice. | [21] |
|  | In mice, G-CSF pathway regulates anti-dsDNA antibodies production. | [22] |
|  | G-CSF, as a grow factor for neutrophils, may contribute on NETosis formation. | [23] |
| **IFNα** | IFNα-induced gene signature is considered a hallmark of SLE. | [24–26] |
|  | Chronic dysregulation of plasmacytoid dendritic cells (pDC) in SLE mice models induced increase IFNα secretion by dendritic cells.  When pDC internalize NETs, which contain endogenous DNA and neutrophil proteins, they are activated and secrete IFNα | [27–30] |
|  | Associated with increased of plasma cells numbers, autoantibody production, defective apoptotic cell clearance, and promotion of T-cell-dependent inflammation. High levels are observed in first degree relatives of SLE patients, which may suggest a genetic background for susceptibility.  IFNα together with IFNλ1 define subsets of patients with SLE with different characteristics. High disease activity is associated with either simultaneous upregulation of IFNλ1 and IFNα. | [28,31–33] |
|  | In mice studies, blockade of IFNα secretion or pathway is associated with better outcomes. | [34–36] |
|  | Targeted therapy (i.e., sifalimumab, anifrolumab) showed promising results. | [37–39] |
| **TNFα** | Controversial role. In some mouse models, it appears to have immunomodulatory role inducing less severity, but in others it can be associated with deleterious effects. | [40–45] |
|  | In some studies, TNFα levels were found to be increased and correlated with disease activity. In others, no differences between patients and controls were found. One study reported inverse association between the concentration of TNFα in SLE patients and severity of the disease. | [12,16,46–48] |
|  | Anti-dsDNA antibodies enhance the release and expression of TNFα mRNA from human mononuclear cells. | [3] |
|  | Polymorphisms in TNFα promoter is associated with SLE risk. | [49] |
|  | TNFα antagonists induced SLE-like disease, suggesting TNFα is beneficial in SLE context. | [50] |
| **IL-12** | Levels are high in patients as compared to controls and correlate with anti-dsDNA antibodies. Levels are normalized with corticosteroid treatment. | [12,14,47,51] |
|  | Ab to ribosomal P proteins induce Th1 responses, by upregulating the production of IL-12 by activated monocytes. Therefore, anti-ribosomal P Ab play an important role in the pathogenesis of SLE through the promotion of Th1 responses. | [52] |
|  | Serum p40 monomers (but not IL-12 p70 titers) are elevated in the sera of patients and correlate with disease activity. | [53] |
|  | IL-12B genetic variants are risk factors for SLE and predict disease phenotype. | [54,55] |
| **IL-23** | *In vitro* IL-23 treatment promotes IL-17 production and downregulates IL-2 production. The IL-23R knockdown mouse-model presents fewer T follicular helper cells, B cells, and plasma cells, leading to decreased production of anti-dsDNA antibodies. Therefore, IL-23 accounts for the main aspects of human and murine lupus, including the expansion of double negative T cells, decreased IL-2, and increased IL-17 production. | [56,57] |
|  | Loss of IL-23/IL-17 by genetic deletion of IL-23/p19 in the Ro52(-/-) mice conferred protection from skin disease and systemic autoimmunity. These results reveal that the lupus-associated Ro52 protein is an important negative regulator of pro-inflammatory cytokine production, and they provide a mechanism by which a defective Ro52 function can lead to tissue inflammation and systemic autoimmunity through the IL-23-Th17 pathway. | [58] |
|  | Mice treated with a neutralizing anti–IL-23 antibody had less severe nephritis than control-treated mice, suggesting that IL-23 plays a role in the development of autoimmunity and ensuing inflammation. | [59] |
|  | IL-23 has a role in the development and pathogenesis of disease. Patients with active disease showed higher IL-23 mRNAs compared with patients with inactive disease as well as healthy controls. IL-23 levels were significantly higher in patients with renal involvement. Conversely, expression of mRNA IL-23 in biopsies of patients with lupus nephritis was shown to be inversely correlated with histological disease activity index. | [60–62] |
|  | IL23R polymorphisms may be involved in susceptibility to SLE. | [63] |
|  | Increased levels have been found in SLE and clinical trials with anti-IL-12/23 are underway. | [64] |
| **IL-10** | Serum levels are elevated and correlate with disease activity (SLEDAI) and production of anti-dsDNA antibodies. | [65–72] |
|  | In mice, this cytokine may play either a promoting or protective role. Administration of anti-IL-10 antibody could significantly delay the development of lupus in New Zealand mice model. IL-10 blockade limited the renal damage in murine models of lupus nephritis-severe combined immunodeficient, and a decrease in the production of IgG anti-DNA antibodies. Contrarily, studies also support a protective role of IL-10 by down-regulating pathogenic Th1 responses and inhibiting pathogenic IFNγ production in mice models. | [73–76] |
|  | Overproduction of IL-10 in SLE patients contributed to the pathogenesis of this disease, promoting the high titer production of autoantibodies by B cells hyperactivity. In addition, CD4+ICOS+FoxP3+ T lymphocytes also can produce a large amount of IL-10 in SLE patients, which further control Th17 cells in an IL-10-dependent manner. | [77–80] |
|  | Down-modulation and polymorphisms in the IL-10 receptor gene appear to be present in SLE patients, which would account for an impaired IL-10 function. | [71] |
| **IL-17** | Levels of IL-17A are increased in different types of SLE patients (pediatric, juvenile or pregnant women). Moreover, IL-17A mRNA expression and serum levels correlate positively with disease activity. In addition, IL-17 levels have also been observed in skin and central nervous system lesions. In contrast, expression of mRNA IL-17 and RORγin biopsies of patients with lupus nephritis was shown to be inversely correlated with histological disease activity index. | [62,81–91] |
|  | High levels observed in lupus murine models. Deregulated production of IL-17 and IL-21 can lead to lupus-like disease symptoms, based on genetic background. Also, high levels of IL-17 were observed in a different lupus model induced by activated lymphocyte derived DNA. | [90,92] |
|  | Promotes the inflammatory process by inducing the local production of chemokines and cytokines from several cell types (e.g., epithelial cells and fibroblasts). It can amplify the immune response by augmenting the production of autoantibodies through stimulation of B lymphocytes.  Expression level was associated with immune complex deposition and complement activation in kidney. | [93–95] |
| **IL1β** | Mouse model knocked down for IL-1β gene are less susceptible to Lupus. | [96] |
|  | Highly expressed in kidneys of mice with lupus nephritis and is generated by glomerular macrophages. | [97] |
|  | Polymorphism in gene encoding for IL1β is associated with SLE. | [98–100] |
|  | Related to inflammasome activation, which has been proposed as a mechanism in SLE pathophysiology. Inflammasome activation in SLE presents several mechanisms, such as stimulation of Toll-Like Receptors and NF κβ transcription by immune complexes or C3a. | [101] |
| **IL-2** | IL-2/IL-2R deficient mice present SLE hallmarks such a as autoantibody production, lymphadenopathy, and decreased T regulatory cells. | [102] |
|  | Low levels of IL-2 have been observed in SLE patients, triggering low counts of CD4 T regulatory cells | [103–105] |
|  | Clinical trials with IL-2 therapy are underway and results are promising. | [106–108] |
| **IL-4** | IL-4 role in rescuing B-cell from apoptosis may promote autoreactive B-cells survival in mouse model. Indeed, IL-4 treatment trigger the production of IgG anti-dsDNA antibodies and blockade of IL-4 prevent the upset of lupus nephritis. | [109–111] |
|  | In mouse model, described as protective due to its Ig isotype switching role from pathogenic subclass (i.e., IgG2 or IgG3) to less pathogenic IgG1 one. Indeed, IgG1 anti-dsDNA antibodies are known to be less pathologic and less able to induce nephritis. | [112,113] |
|  | Inhibits T-cell-dependent Ab responses and tissue inflammation. Thus, decrease on CD4-T-cells producing IL-4 is associated with increase in the activity of the disease. | [113,114] |
|  | In patients IL-4 levels are lower than in healthy controls. | [12,115] |
| **IL-13** | Elevated in patients with active lupus nephritis. Moreover, increased CD38 expression in SLE T cells correlated with plasma levels of IL-13. A study found high levels in different ADs, including SLE, when compared to controls. However, a study using multiplex array found no difference in serum concentrations between SLE patients with lupus nephritis and healthy controls. | [116–120] |
|  | Levels were correlated with disease activity (SLEDAI), erythrocyte sedimentation rate and serum levels of C3. | [121] |
|  | T cells infiltrating the glomeruli and perivascular areas predominantly produced IFNγ, IL-13, and IL-17 in the MRL/lpr, B/W F1, and BXSB lupus models. Thus, IL-13 may also be an important factor in the pathogenesis of glomerulonephritis and vasculitis. | [122] |
|  | DNA methylation levels within *IL10* and *IL13* gene regulatory domains are reduced in SLE CD4+ T cells relative to healthy controls and negatively correlate with IL-10 and IL-13 mRNA expression. | [123] |
|  | Levels were correlated with RF in SLE, rheumatoid arthritis and systemic sclerosis. Moreover, an association with anti-Ro was observed in Sjögren’s syndrome. No association with anti-dsDNA, Sm, RNP, La or Scl70 antibodies was detected. | [117] |
| **IL-9** | mRNA and serum levels of IL-9 are elevated in patients. Moreover, IL-9 levels on serum and percentages of Th9 cells correlate with the SLE disease activity and are associated with proteinuria and low C3 titers. Active patients, who were treated and achieved disease control, disclosed a reduction in IL-9. A close relationship between IL-9/Th9 and Th17 is suspected, due to a positive feedback loop between these sub-populations. | [124–129] |
|  | Lupus-prone mice models have shown an increase in production of IL-9 and expansion of Th9 lymphocytes, which were associated with anti-dsDNA antibodies titers. In addition, IL-9 appears to promote B-lymphocytes proliferation and autoantibodies production, which could be blocked by inhibition of signal transducer STAT3. IL-9 blockade reduced serum anti-dsDNA antibodies titers and lessened renal disease in this mice model. | [130] |
| **IFNγ** | Elevated in SLE patient’s serum and in murine models of SLE. Its blockade abrogates SLE development in mice. | [12,115,131–135] |
|  | Current anti-IFNγ monoclonal antibody (AMG811) is under clinical study, with no clinical benefits observed. | [26] |
| **IL-5** | Studies have disclosed contradictory results concerning its serum levels | [136,137] |
|  | Patients with severe/extensive skin lesions showed an overexpression of IL-5. These results suggest that Th2 cells are involved in SLE skin inflammation | [138] |
|  | High expression of IL-5 in SLE-prone mice may directly or indirectly mediate a skewed signaling of proliferation/differentiation of self-antigen-activated B1 cells, leading to suppression of autoimmune disease | [139] |

**References for Additional file 4. Table S2.**

1. Rovin BH, Lu L, Zhang X. A novel interleukin-8 polymorphism is associated with severe systemic lupus erythematosus nephritis. Kidney Int. 2002;62:261–5.

2. Yung S, Ng CYC, Au KY, Cheung KF, Zhang Q, Zhang C, et al. Binding of anti-dsDNA antibodies to proximal tubular epithelial cells contributes to renal tubulointerstitial inflammation. Clin. Sci. 2017;131:49–67.

3. Sun KH, Yu CL, Tang SJ, Sun GH. Monoclonal anti-double-stranded DNA autoantibody stimulates the expression and release of IL-1beta, IL-6, IL-8, IL-10 and TNF-alpha from normal human mononuclear cells involving in the lupus pathogenesis. Immunology. 2000;99:352–60.

4. Yao Y, Wang JB, Xin MM, Li H, Liu B, Wang LL, et al. Balance between inflammatory and regulatory cytokines in systemic lupus erythematosus. Genet. Mol. Res. 2016;15:1–8.

5. Yoshio T, Okamoto H, Kurasawa K, Dei Y, Hirohata S, Minota S. IL-6, IL-8, IP-10, MCP-1 and G-CSF are significantly increased in cerebrospinal fluid but not in sera of patients with central neuropsychiatric lupus erythematosus. Lupus. 2016;25:997–1003.

6. Wu Y, Cai B, Zhang J, Shen B, Huang Z, Tan C, et al. IL-1β and IL-6 Are Highly Expressed in RF+IgE+ Systemic Lupus Erythematous Subtype. J. Immunol. Res. 2017;2017:5096741.

7. López P, Rodríguez-Carrio J, Martínez-Zapico A, Caminal-Montero L, Suarez A. Senescent profile of angiogenic T cells from systemic lupus erythematosus patients. J. Leukoc. Biol. 2016;99:405–12.

8. Vega L, Barbado J, Almansa R, González-Gallego R, Rico L, Jimeno A, et al. Prolonged standard treatment for systemic lupus erythematosus fails to normalize the secretion of innate immunity-related chemokines. Eur. Cytokine Netw. 2010;21:71–6.

9. El-Shehaby A, Darweesh H, El-Khatib M, Momtaz M, Marzouk S, El-Shaarawy N, et al. Correlations of urinary biomarkers, TNF-like weak inducer of apoptosis (TWEAK), osteoprotegerin (OPG), monocyte chemoattractant protein-1 (MCP-1), and IL-8 with lupus nephritis. J. Clin. Immunol. 2011;31:848–56.

10. Gupta AK, Giaglis S, Hasler P, Hahn S. Efficient neutrophil extracellular trap induction requires mobilization of both intracellular and extracellular calcium pools and is modulated by cyclosporine A. PLoS One. 2014;9:e97088.

11. Remijsen Q, Kuijpers TW, Wirawan E, Lippens S, Vandenabeele P, Vanden Berghe T. Dying for a cause: NETosis, mechanisms behind an antimicrobial cell death modality. Cell Death Differ. 2011;18:581–8.

12. Guimarães PM, Scavuzzi BM, Stadtlober NP, Franchi Santos LF da R, Lozovoy MAB, Iriyoda TMV, et al. Cytokines in systemic lupus erythematosus: far beyond Th1/Th2 dualism lupus: cytokine profiles. Immunol. Cell Biol. 2017;doi:10.1038/icb.2017.53.

13. Cigni A, Pileri PV, Faedda R, Gallo P, Sini A, Satta AE, et al. Interleukin 1, interleukin 6, interleukin 10, and tumor necrosis factor α in active and quiescent systemic lupus erythematosus. J. Investig. Med. 2014;62:825–9.

14. Talaat RM, Mohamed SF, Bassyouni IH, Raouf AA. Th1/Th2/Th17/Treg cytokine imbalance in systemic lupus erythematosus (SLE) patients: Correlation with disease activity. Cytokine. 2015;72:146–53.

15. Eilertsen GØ, Nikolaisen C, Becker-Merok A, Nossent JC. Interleukin-6 promotes arthritis and joint deformation in patients with systemic lupus erythematosus. Lupus. 2011;20:607–13.

16. Umare V, Pradhan V, Nadkar M, Rajadhyaksha A, Patwardhan M, Ghosh KK, et al. Effect of proinflammatory cytokines (IL-6, TNF-α, and IL-1β) on clinical manifestations in Indian SLE patients. Mediators Inflamm. 2014;2014:385297.

17. Illei GG, Shirota Y, Yarboro CH, Daruwalla J, Tackey E, Takada K, et al. Tocilizumab in systemic lupus erythematosus: data on safety, preliminary efficacy, and impact on circulating plasma cells from an open-label phase I dosage-escalation study. Arthritis Rheum. 2010;62:542–52.

18. Menon M, Blair PA, Isenberg DA, Mauri C. A Regulatory Feedback between Plasmacytoid Dendritic Cells and Regulatory B Cells Is Aberrant in Systemic Lupus Erythematosus. Immunity. 2016;44:683–97.

19. Cui YX, Fu CW, Jiang F, Ye LX, Meng W. Association of the interleukin-6 polymorphisms with systemic lupus erythematosus: a meta-analysis. Lupus. 2015;24:1308–17.

20. Zavala F, Masson A, Hadaya K, Ezine S, Schneider E, Babin O, et al. Granulocyte-colony stimulating factor treatment of lupus autoimmune disease in MRL-lpr/lpr mice. J. Immunol. 1999;163:5125–32.

21. Yan JJ, Jambaldorj E, Lee JG, Jang JY, Shim JM, Han M, et al. Granulocyte colony-stimulating factor treatment ameliorates lupus nephritis through the expansion of regulatory T cells. BMC Nephrol. 2016;17:175.

22. Lantow M, Sivakumar R, Zeumer L, Wasserfall C, Zheng Y-Y, Atkinson MA, et al. The granulocyte colony stimulating factor pathway regulates autoantibody production in a murine induced model of systemic lupus erythematosus. Arthritis Res. Ther. 2013;15:R49.

23. Tsokos GC, Lo MS, Costa Reis P, Sullivan KE. New insights into the immunopathogenesis of systemic lupus erythematosus. Nat. Rev. Rheumatol. 2016;12:716–30.

24. Bennett L, Palucka AK, Arce E, Cantrell V, Borvak J, Banchereau J, et al. Interferon and granulopoiesis signatures in systemic lupus erythematosus blood. J. Exp. Med. 2003;197:711–23.

25. Obermoser G, Pascual V. The interferon-alpha signature of systemic lupus erythematosus. Lupus. 2010;19:1012–9.

26. Mathian A, Hie M, Cohen-Aubart F, Amoura Z. Targeting interferons in systemic lupus erythematosus: current and future prospects. Drugs. 2015;75:835–46.

27. Rönnblom L, Eloranta ML. The interferon signature in autoimmune diseases. Curr. Opin. Rheumatol. 2013;25:248–53.

28. Li H, Fu YX, Wu Q, Zhou Y, Crossman DK, Yang P, et al. Interferon-induced mechanosensing defects impede apoptotic cell clearance in lupus. J. Clin. Invest. 2015;125:2877–90.

29. Garcia-Romo GS, Caielli S, Vega B, Connolly J, Allantaz F, Xu Z, et al. Netting neutrophils are major inducers of type I IFN production in pediatric systemic lupus erythematosus. Sci. Transl. Med. 2011;3:73ra20.

30. Lande R, Ganguly D, Facchinetti V, Frasca L, Conrad C, Gregorio J, et al. Neutrophils activate plasmacytoid dendritic cells by releasing self-DNA-peptide complexes in systemic lupus erythematosus. Sci. Transl. Med. 2011;3:73ra19.

31. Pascual V, Farkas L, Banchereau J. Systemic lupus erythematosus: all roads lead to type I interferons. Curr. Opin. Immunol. 2006;18:676–82.

32. Brodin P, Davis MM. Human immune system variation. Nat. Rev. Immunol. 2017;17:21–9.

33. Oke V, Brauner S, Larsson A, Gustafsson J, Zickert A, Gunnarsson I, et al. IFN-λ1 with Th17 axis cytokines and IFN-α define different subsets in systemic lupus erythematosus (SLE). Arthritis Res. Ther. 2017;19:139 doi: 10.1186/s13075-017-1344-7.

34. Rowland SL, Riggs JM, Gilfillan S, Bugatti M, Vermi W, Kolbeck R, et al. Early, transient depletion of plasmacytoid dendritic cells ameliorates autoimmunity in a lupus model. J. Exp. Med. 2014;211:1977–91.

35. Baccala R, Gonzalez-Quintial R, Schreiber RD, Lawson BR, Kono DH, Theofilopoulos AN. Anti-IFN-α/β receptor antibody treatment ameliorates disease in lupus-predisposed mice. J. Immunol. 2012;189:5976–84.

36. Baccala R, Gonzalez-Quintial R, Blasius AL, Rimann I, Ozato K, Kono DH, et al. Essential requirement for IRF8 and SLC15A4 implicates plasmacytoid dendritic cells in the pathogenesis of lupus. Proc. Natl. Acad. Sci. U. S. A. 2013;110:2940–5.

37. Zharkova O, Celhar T, Cravens PD, Satterthwaite AB, Fairhurst A-M, Davis LS. Pathways leading to an immunological disease: systemic lupus erythematosus. Rheumatology. 2017;56:i55–66.

38. Furie R, Khamashta M, Merrill JT, Werth VP, Kalunian K, Brohawn P, et al. Anifrolumab, an Anti-Interferon-α Receptor Monoclonal Antibody, in Moderate-to-Severe Systemic Lupus Erythematosus. Arthritis Rheumatol. 2017;69:376–86.

39. Khamashta M, Merrill JT, Werth VP, Furie R, Kalunian K, Illei GG, et al. Sifalimumab, an anti-interferon-α monoclonal antibody, in moderate to severe systemic lupus erythematosus: a randomised, double-blind, placebo-controlled study. Ann. Rheum. Dis. 2016;75:1909–16.

40. Jacob CO, McDevitt HO. Tumour necrosis factor-alpha in murine autoimmune “lupus” nephritis. Nature. 1988;331:356–8.

41. Gordon C, Ranges GE, Greenspan JS, Wofsy D. Chronic therapy with recombinant tumor necrosis factor-alpha in autoimmune NZB/NZW F1 mice. Clin. Immunol. Immunopathol. 1989;52:421–34.

42. Gordon C, Wofsy D. Effects of recombinant murine tumor necrosis factor-alpha on immune function. J. Immunol. 1990;144:1753–8.

43. Kontoyiannis D, Kollias G. Accelerated autoimmunity and lupus nephritis in NZB mice with an engineered heterozygous deficiency in tumor necrosis factor. Eur. J. Immunol. 2000;30:2038–47.

44. Boswell JM, Yui MA, Burt DW, Kelley VE. Increased tumor necrosis factor and IL-1 beta gene expression in the kidneys of mice with lupus nephritis. J. Immunol. 1988;141:3050–4.

45. Yokoyama H, Kreft B, Kelley VR. Biphasic increase in circulating and renal TNF-alpha in MRL-lpr mice with differing regulatory mechanisms. Kidney Int. 1995;47:122–30.

46. Postal M, Appenzeller S. The role of Tumor Necrosis Factor-alpha (TNF-α) in the pathogenesis of systemic lupus erythematosus. Cytokine. 2011;56:537–43.

47. McCarthy EM, Smith S, Lee RZ, Cunnane G, Doran MF, Donnelly S, et al. The association of cytokines with disease activity and damage scores in systemic lupus erythematosus patients. Rheumatology. 2014;53:1586–94.

48. Gómez D, Correa PA, Gómez LM, Cadena J, Molina JF, Anaya J-M. Th1/Th2 cytokines in patients with systemic lupus erythematosus: is tumor necrosis factor alpha protective? Semin. Arthritis Rheum. 2004;33:404–13.

49. Lee YH, Harley JB, Nath SK. Meta-analysis of TNF-alpha promoter -308 A/G polymorphism and SLE susceptibility. Eur. J. Hum. Genet. 2006;14:364–71.

50. Mudduluru BM, Shah S, Shamah S, Swaminath A. TNF-alpha antagonist induced lupus on three different agents. Postgrad. Med. 2017;129:304–6.

51. Qiu F, Song L, Yang N, Li X. Glucocorticoid downregulates expression of IL-12 family cytokines in systemic lupus erythematosus patients. Lupus. 2013;22:1011–6.

52. Nagai T, Yanagida T, Hirohata S. Anti-ribosomal P protein antibody induces Th1 responses by enhancing the production of IL-12 in activated monocytes. Mod. Rheumatol. 2011;21:57–62.

53. Lauwerys BR, Van Snick J, Houssiau FA. Serum IL-12 in systemic lupus erythematosus: absence of p70 heterodimers but presence of p40 monomers correlating with disease activity. Lupus. 2002;11:384–7.

54. Paradowska-Gorycka A, Sowinska A, Stypinska B, Grobelna MK, Walczyk M, Olesinska M, et al. Genetic Variants in IL-12B and IL-27 in the Polish Patients with Systemic Lupus Erythematosus. Scand. J. Immunol. 2016;84:49–60.

55. Miteva LD, Manolova IM, Ivanova MG, Rashkov RK, Stoilov RM, Gulubova M V, et al. Functional genetic polymorphisms in interleukin-12B gene in association with systemic lupus erythematosus. Rheumatol. Int. 2012;32:53–9.

56. Dai H, He F, Tsokos GC, Kyttaris VC. IL-23 Limits the Production of IL-2 and Promotes Autoimmunity in Lupus. J. Immunol. 2017;199:903–10.

57. Shaltout AS, Sayed D, Badary MS, Nafee AM, El Zohri MH, Bakry R, et al. Effect of IL6 and IL23 on double negative T cells and anti ds-DNA in systemic lupus erythematosus patients. Hum. Immunol. 2016;77:937–43.

58. Espinosa A, Dardalhon V, Brauner S, Ambrosi A, Higgs R, Quintana FJ, et al. Loss of the lupus autoantigen Ro52/Trim21 induces tissue inflammation and systemic autoimmunity by disregulating the IL-23-Th17 pathway. J. Exp. Med. 2009;206:1661–71.

59. Kyttaris VC, Kampagianni O, Tsokos GC. Treatment with anti-interleukin 23 antibody ameliorates disease in lupus-prone mice. Biomed Res. Int. 2013;2013:861028.

60. ElGlil RR, Emam SM, Hashaad NI. Expression of IL-23 mRNA in Systemic Lupus Erythematosus Patients: Relation with Disease Activity. Egypt. J. Immunol. 2015;22:9–21.

61. Du J, Li Z, Shi J, Bi L. Associations between serum interleukin-23 levels and clinical characteristics in patients with systemic lupus erythematosus. J. Int. Med. Res. 2014;42:1123–30.

62. Kwan BCH, Tam LS, Lai KB, Lai FMM, Li EKM, Wang G, et al. The gene expression of type 17 T-helper cell-related cytokines in the urinary sediment of patients with systemic lupus erythematosus. Rheumatology. 2009;48:1491–7.

63. Paradowska-Gorycka A, Sowinska A, Stypinska B, Grobelna MK, Walczyk M, Olesinska M, et al. Impact of the IL-17F, IL-23 and IL-23R on susceptibility and phenotype of systemic lupus erythematosus. Autoimmunity. 2016;49:373–82.

64. Teng MWL, Bowman EP, McElwee JJ, Smyth MJ, Casanova JL, Cooper AM, et al. IL-12 and IL-23 cytokines: from discovery to targeted therapies for immune-mediated inflammatory diseases. Nat. Med. 2015;21:719–29.

65. Park YB, Lee SK, Kim DS, Lee J, Lee CH, Song CH. Elevated interleukin-10 levels correlated with disease activity in systemic lupus erythematosus. Clin. Exp. Rheumatol. 1998;16:283–8.

66. Gelati M, Lamperti E, Dufour A, Corsini E, Venegoni E, Milanese C, et al. IL-10 production in multiple sclerosis patients, SLE patients and healthy controls: preliminary findings. Ital. J. Neurol. Sci. 1997;18:191–4.

67. Chun HY, Chung JW, Kim HA, Yun JM, Jeon JY, Ye YM, et al. Cytokine IL-6 and IL-10 as biomarkers in systemic lupus erythematosus. J. Clin. Immunol. 2007;27:461–6.

68. Mellor-Pita S, Citores MJ, Castejon R, Yebra-Bango M, Tutor-Ureta P, Rosado S, et al. Monocytes and T lymphocytes contribute to a predominance of interleukin 6 and interleukin 10 in systemic lupus erythematosus. Cytometry B. Clin. Cytom. 2009;76:261–70.

69. Houssiau FA, Lefebvre C, Vanden Berghe M, Lambert M, Devogelaer JP, Renauld JC. Serum interleukin 10 titers in systemic lupus erythematosus reflect disease activity. Lupus. 1995;4:393–5.

70. El-Sayed M, Nofal E, al Mokadem S, al Makhzangy I, Gaballah H, Akl H. Correlative study of serum Th1/Th2 cytokines levels in patients with systemic lupus erythematosus with SLEDAI. Egypt Dermatol Online J. 2008;4:3–19.

71. Peng H, Wang W, Zhou M, Li R, Pan HF, Ye DQ. Role of interleukin-10 and interleukin-10 receptor in systemic lupus erythematosus. Clin. Rheumatol. 2013;32:1255–66.

72. El-Fetouh SA, Mohammed RA, Abozaid Mohmad HS. Serum interleukin-18 and interleukin-10 levels in systemic lupus erythematosus: correlation with SLEDAI score and disease activity parameters. Egypt. Rheumatol. Rehabil. 2014;41:160.

73. Ishida H, Muchamuel T, Sakaguchi S, Andrade S, Menon S, Howard M. Continuous administration of anti-interleukin 10 antibodies delays onset of autoimmunity in NZB/W F1 mice. J. Exp. Med. 1994;179:305–10.

74. Ravirajan CT, Wang Y, Matis LA, Papadaki L, Griffiths MH, Latchman DS, et al. Effect of neutralizing antibodies to IL-10 and C5 on the renal damage caused by a pathogenic human anti-dsDNA antibody. Rheumatology. 2004;43:442–7.

75. Ling GS, Cook HT, Botto M, Lau YL, Huang FP. An essential protective role of IL-10 in the immunological mechanism underlying resistance vs. susceptibility to lupus induction by dendritic cells and dying cells. Rheumatology. 2011;50:1773–84.

76. Yin Z, Bahtiyar G, Zhang N, Liu L, Zhu P, Robert ME, et al. IL-10 regulates murine lupus. J. Immunol. 2002;169:2148–55.

77. Llorente L, Zou W, Levy Y, Richaud-Patin Y, Wijdenes J, Alcocer-Varela J, et al. Role of interleukin 10 in the B lymphocyte hyperactivity and autoantibody production of human systemic lupus erythematosus. J. Exp. Med. 1995;181:839–44.

78. Lauwerys BR, Garot N, Renauld JC, Houssiau FA. Interleukin-10 blockade corrects impaired in vitro cellular immune responses of systemic lupus erythematosus patients. Arthritis Rheum. 2000;43:1976–81.

79. Liu Y, Zhu T, Cai G, Qin Y, Wang W, Tang G, et al. Elevated circulating CD4+ ICOS+ Foxp3+ T cells contribute to overproduction of IL-10 and are correlated with disease severity in patients with systemic lupus erythematosus. Lupus. 2011;20:620–7.

80. Zhao X-F, Pan H-F, Yuan H, Zhang W-H, Li X-P, Wang G-H, et al. Increased serum interleukin 17 in patients with systemic lupus erythematosus. Mol. Biol. Rep. 2010;37:81–5.

81. Yu B, Guan M, Peng Y, Shao Y, Zhang C, Yue X, et al. Copy number variations of interleukin-17F, interleukin-21, and interleukin-22 are associated with systemic lupus erythematosus. Arthritis Rheum. 2011;63:3487–92.

82. Bălănescu P, Bălănescu E, Tănăsescu C, Nicolau A, Tănăsescu R, Grancea C, et al. T helper 17 cell population in lupus erythematosus. Rom. J. Intern. Med. 2010;48:255–9.

83. Rana A, Minz RW, Aggarwal R, Anand S, Pasricha N, Singh S. Gene expression of cytokines (TNF-α, IFN-γ), serum profiles of IL-17 and IL-23 in paediatric systemic lupus erythematosus. Lupus. 2012;21:1105–12.

84. Torricelli M, Bellisai F, Novembri R, Galeazzi LR, Iuliano A, Voltolini C, et al. High levels of maternal serum IL-17 and activin A in pregnant women affected by systemic lupus erythematosus. Am. J. Reprod. Immunol. 2011;66:84–9.

85. Chen XQ, Yu YC, Deng HH, Sun JZ, Dai Z, Wu YW, et al. Plasma IL-17A is increased in new-onset SLE patients and associated with disease activity. J. Clin. Immunol. 2010;30:221–5.

86. Abou Ghanima AT, Elolemy GG, Ganeb SS, Abo Elazem AA, Abdelgawad ER. Role of T helper 17 cells in the pathogenesis of systemic lupus erythematosus. Egypt. J. Immunol. 2012;19:25–33.

87. Tanasescu C, Balanescu E, Balanescu P, Olteanu R, Badea C, Grancea C, et al. IL-17 in cutaneous lupus erythematosus. Eur. J. Intern. Med. 2010;21:202–7.

88. Lu XY, Zhu CQ, Qian J, Chen XX, Ye S, Gu YY. Intrathecal cytokine and chemokine profiling in neuropsychiatric lupus or lupus complicated with central nervous system infection. Lupus. 2010;19:689–95.

89. Chen DY, Chen YM, Wen MC, Hsieh TY, Hung WT, Lan JL. The potential role of Th17 cells and Th17-related cytokines in the pathogenesis of lupus nephritis. Lupus. 2012;21:1385–96.

90. Wen Z, Xu L, Xu W, Yin Z, Gao X, Xiong S. Interleukin-17 expression positively correlates with disease severity of lupus nephritis by increasing anti-double-stranded DNA antibody production in a lupus model induced by activated lymphocyte derived DNA. PLoS One. 2013;8:e58161.

91. Koga T, Ichinose K, Tsokos GC. T cells and IL-17 in lupus nephritis. Clin. Immunol. 2016;doi: 10.1016/j.clim.2016.04.010.

92. Biswas PS, Kang K, Gupta S, Bhagat G, Pernis AB. A murine autoimmune model of rheumatoid arthritis and systemic lupus erythematosus associated with deregulated production of IL-17 and IL-21. Methods Mol. Biol. 2012;900:233–51.

93. Li D, Guo B, Wu H, Tan L, Chang C, Lu Q. Interleukin-17 in systemic lupus erythematosus: A comprehensive review. Autoimmunity. 2015;48:353–61.

94. Doreau A, Belot A, Bastid J, Riche B, Trescol-Biemont M-C, Ranchin B, et al. Interleukin 17 acts in synergy with B cell-activating factor to influence B cell biology and the pathophysiology of systemic lupus erythematosus. Nat. Immunol. 2009;10:778–85.

95. Ahmed S, Anolik JH. B-cell biology and related therapies in systemic lupus erythematosus. Rheum. Dis. Clin. North Am. 2010;36:109–30, viii–ix.

96. Voronov E, Dayan M, Zinger H, Gayvoronsky L, Lin J-P, Iwakura Y, et al. IL-1 beta-deficient mice are resistant to induction of experimental SLE. Eur. Cytokine Netw. 2006;17:109–16.

97. Boswell JM, Yui MA, Endres S, Burt DW, Kelley VE. Novel and enhanced IL-1 gene expression in autoimmune mice with lupus. J. Immunol. 1988;141:118–24.

98. Mohammadoo-Khorasani M, Salimi S, Tabatabai E, Sandoughi M, Zakeri Z, Farajian-Mashhadi F. Interleukin-1β (IL-1β) & IL-4 gene polymorphisms in patients with systemic lupus erythematosus (SLE) & their association with susceptibility to SLE. Indian J. Med. Res. 2016;143:591–6.

99. Song GG, Kim J-H, Seo YH, Choi SJ, Ji JD, Lee YH. Associations between interleukin 1 polymorphisms and susceptibility to systemic lupus erythematosus: a meta-analysis. Hum. Immunol. 2014;75:105–12.

100. Camargo JF, Correa PA, Castiblanco J, Anaya J-M. Interleukin-1beta polymorphisms in Colombian patients with autoimmune rheumatic diseases. Genes Immun. 2004;5:609–14.

101. Kahlenberg JM, Kaplan MJ. The inflammasome and lupus: another innate immune mechanism contributing to disease pathogenesis? Curr. Opin. Rheumatol. 2014;26:475–81.

102. Schorle H, Holtschke T, Hünig T, Schimpl A, Horak I. Development and function of T cells in mice rendered interleukin-2 deficient by gene targeting. Nature. 1991;352:621–4.

103. Solomou EE, Juang YT, Gourley MF, Kammer GM, Tsokos GC. Molecular basis of deficient IL-2 production in T cells from patients with systemic lupus erythematosus. J. Immunol. 2001;166:4216–22.

104. Kovacs B, Vassilopoulos D, Vogelgesang SA, Tsokos GC. Defective CD3-mediated cell death in activated T cells from patients with systemic lupus erythematosus: role of decreased intracellular TNF-alpha. Clin. Immunol. Immunopathol. 1996;81:293–302.

105. Xu L, Zhang L, Yi Y, Kang H-K, Datta SK. Human lupus T cells resist inactivation and escape death by upregulating COX-2. Nat. Med. 2004;10:411–5.

106. Humrich JY, Riemekasten G. Restoring regulation - IL-2 therapy in systemic lupus erythematosus. Expert Rev. Clin. Immunol. 2016;12:1153–60.

107. Mizui M, Tsokos GC. Low-Dose IL-2 in the Treatment of Lupus. Curr. Rheumatol. Rep. 2016;18:68.

108. He J, Zhang X, Wei Y, Sun X, Chen Y, Deng J, et al. Low-dose interleukin-2 treatment selectively modulates CD4(+) T cell subsets in patients with systemic lupus erythematosus. Nat. Med. 2016;22:991–3.

109. Erb KJ, Rüger B, von Brevern M, Ryffel B, Schimpl A, Rivett K. Constitutive expression of interleukin (IL)-4 in vivo causes autoimmune-type disorders in mice. J. Exp. Med. 1997;185:329–39.

110. Nakajima A, Hirose S, Yagita H, Okumura K. Roles of IL-4 and IL-12 in the development of lupus in NZB/W F1 mice. J. Immunol. 1997;158:1466–72.

111. Deocharan B, Marambio P, Edelman M, Putterman C. Differential effects of interleukin-4 in peptide induced autoimmunity. Clin. Immunol. 2003;108:80–8.

112. Peng SL, Moslehi J, Craft J. Roles of interferon-gamma and interleukin-4 in murine lupus. J. Clin. Invest. 1997;99:1936–46.

113. Singh RR. IL-4 and many roads to lupuslike autoimmunity. Clin. Immunol. 2003;108:73–9.

114. Sugimoto K, Morimoto S, Kaneko H, Nozawa K, Tokano Y, Takasaki Y, et al. Decreased IL-4 producing CD4+ T cells in patients with active systemic lupus erythematosus-relation to IL-12R expression. Autoimmunity. 2002;35:381–7.

115. Elewa EA, Zakaria O, Mohamed EI, Boghdadi G. The role of interleukins 4, 17 and interferon gamma as biomarkers in patients with Systemic Lupus Erythematosus and their correlation with disease activity. Egypt. Rheumatol. 2014;36:21–7.

116. Chen X, Zhang Z, Jiang L, Ye F, Wang J, Wu P. Elevated interleukin-13 in patients with active lupus nephritis. Chin. Med. J. (Engl). 2001;114:1022–5.

117. Spadaro A, Rinaldi T, Riccieri V, Taccari E, Valesini G. Interleukin-13 in autoimmune rheumatic diseases: relationship with the autoantibody profile. Clin. Exp. Rheumatol. 2002;20:213–6.

118. Brugos B, Vincze Z, Sipka S, Szegedi G, Zeher M. Serum and urinary cytokine levels of SLE patients. Pharmazie. 2012;67:411–3.

119. Pavón EJ, Zumaquero E, Rosal-Vela A, Khoo K-M, Cerezo-Wallis D, García-Rodríguez S, et al. Increased CD38 expression in T cells and circulating anti-CD38 IgG autoantibodies differentially correlate with distinct cytokine profiles and disease activity in systemic lupus erythematosus patients. Cytokine. 2013;62:232–43.

120. Sigdel KR, Duan L, Wang Y, Hu W, Wang N, Sun Q, et al. Serum Cytokines Th1, Th2, and Th17 Expression Profiling in Active Lupus Nephritis-IV: From a Southern Chinese Han Population. Mediators Inflamm. 2016;2016:4927530.

121. Xu Z, Chen Y. Determination of serum interleukin-13 and nerve growth factor in patients with systemic lupus erythematosus and clinical significance. J. Huazhong Univ. Sci. Technolog. Med. Sci. 2005;25:360–1.

122. Guo Z, Wang Y, Li R, Huang H, Wang R. Use of laser microdissection in the analysis of renal-infiltrating T cells in murine lupus. Cent. J. Immunol. 2014;39:285–93.

123. Zhao M, Tang J, Gao F, Wu X, Liang Y, Yin H, et al. Hypomethylation of IL10 and IL13 promoters in CD4+ T cells of patients with systemic lupus erythematosus. J. Biomed. Biotechnol. 2010;2010:931018.

124. Ouyang H, Shi Y, Liu Z, Feng S, Li L, Su N, et al. Increased interleukin‑9 and CD4+IL-9+ T cells in patients with systemic lupus erythematosus. Mol. Med. Rep. 2013;7:1031–7.

125. Dantas AT, Marques CDL, da Rocha Junior LF, Cavalcanti MB, Gonçalves SMC, Cardoso PRG, et al. Increased Serum Interleukin-9 Levels in Rheumatoid Arthritis and Systemic Lupus Erythematosus: Pathogenic Role or Just an Epiphenomenon? Dis. Markers. 2015;2015:519638.

126. Pan HF, Leng RX, Li XP, Zheng SG, Ye DQ. Targeting T-helper 9 cells and interleukin-9 in autoimmune diseases. Cytokine Growth Factor Rev. 2013;24:515–22.

127. Leng RX, Pan HF, Ye DQ, Xu Y. Potential roles of IL-9 in the pathogenesis of systemic lupus erythematosus. Am. J. Clin. Exp. Immunol. 2012;1:28–32.

128. Rojas-Zuleta WG, Vásquez G. Th9 lymphocytes: A recent history from IL-9 to its potential role in rheumatic diseases. Autoimmun. Rev. 2016;15:649–55.

129. Ciccia F, Guggino G, Ferrante A, Cipriani P, Giacomelli R, Triolo G. Interleukin-9 and T helper type 9 cells in rheumatic diseases. Clin. Exp. Immunol. 2016;185:125–32.

130. Yang J, Li Q, Yang X, Li M. Interleukin-9 Is Associated with Elevated Anti-Double-Stranded DNA Antibodies in Lupus-Prone Mice. Mol. Med. 2015;21:364–70.

131. Ozmen L, Roman D, Fountoulakis M, Schmid G, Ryffel B, Garotta G. Experimental therapy of systemic lupus erythematosus: the treatment of NZB/W mice with mouse soluble interferon-gamma receptor inhibits the onset of glomerulonephritis. Eur. J. Immunol. 1995;25:6–12.

132. Haas C, Ryffel B, Le Hir M. IFN-gamma is essential for the development of autoimmune glomerulonephritis in MRL/Ipr mice. J. Immunol. 1997;158:5484–91.

133. Amital H, Levi Y, Blank M, Barak V, Langevitz P, Afek A, et al. Immunomodulation of murine experimental SLE-like disease by interferon-gamma. Lupus. 1998;7:445–54.

134. Balomenos D, Rumold R, Theofilopoulos AN. Interferon-gamma is required for lupus-like disease and lymphoaccumulation in MRL-lpr mice. J. Clin. Invest. 1998;101:364–71.

135. Seery JP. IFN-gamma transgenic mice: clues to the pathogenesis of systemic lupus erythematosus? Arthritis Res. 2000;2:437–40.

136. Zhu H, Mi W, Luo H, Chen T, Liu S, Raman I, et al. Whole-genome transcription and DNA methylation analysis of peripheral blood mononuclear cells identified aberrant gene regulation pathways in systemic lupus erythematosus. Arthritis Res. Ther. 2016;18:162.

137. Timóteo RP, Micheli DC, Teodoro RB, Freire M, Bertoncello D, Murta EFC, et al. Characterization of inflammatory markers associated with systemic lupus erythematosus patients undergoing treatment. Rev. Bras. Reumatol. 2016;56:497–503.

138. Carneiro JRM, Fuzii HT, Kayser C, Alberto FL, Soares FA, Sato EI, et al. IL-2, IL-5, TNF-α and IFN-γ mRNA expression in epidermal keratinocytes of systemic lupus erythematosus skin lesions. Clinics. 2011;66:77–82.

139. Wen X, Zhang D, Kikuchi Y, Jiang Y, Nakamura K, Xiu Y, et al. Transgene-mediated hyper-expression of IL-5 inhibits autoimmune disease but increases the risk of B cell chronic lymphocytic leukemia in a model of murine lupus. Eur. J. Immunol. 2004;34:2740–9.
